# Involvement of Metalloproteases in the Fertilization of the Ascidian *Halocynthia roretzi*

**DOI:** 10.3390/biom14121487

**Published:** 2024-11-22

**Authors:** Hitoshi Sawada, Ikuya Hattori, Noritaka Hashii, Takako Saito

**Affiliations:** 1 Sugashima Marine Biological Laboratory, Graduate School of Science, Nagoya University, 429-63 Sugashima, Toba 517-0004, Japan; 2 Department of Food and Nutritional Environment, College of Human Life and Environment, Kinjo Gakuin University, Omori, Moriyama-ku, Nagoya 463-8521, Japan; 3 Division of Biological Chemistry and Biologicals, National Institute of Health Sciences, 3-25-26 Tonomachi, Kawasaki 210-9501, Japan; hashii@nihs.go.jp; 4 Department of Applied Life Sciences, Faculty of Agriculture, Shizuoka University, Shizuoka 422-8529, Japan; saito.takako@shizuoka.ac.jp

**Keywords:** ascidian, fertilization, metalloprotease, astacin, protease, thrombospondin, sperm, lysin, *Halocynthia roretzi*

## Abstract

We previously reported that five astacin-like metalloproteases with thrombospondin type-1 repeats (Tasts) located on the sperm surface are a promising candidate as the protease involved in sperm penetration of the vitelline coat (VC) during fertilization of the ascidian *Ciona intestinalis* type A (Phlebobranchia). However, whether such a protease is involved in the fertilization of other ascidians is unknown. Here, we investigated the effects of four metalloprotease inhibitors on the fertilization of the ascidian *Halocynthia roretzi* (Stolidobranchia). Three metalloprotease inhibitors, GM6001, TAPI-0, and TAPI-1, strongly inhibited fertilization at 33 and 11 μM, whereas TAPI-2 weakly inhibited fertilization at 33 μM. In contrast, GM6001NC (negative control) had no effect on fertilization at 100 μM. Furthermore, GM6001 had no inhibitory effect on the fertilization of VC-deprived eggs. The metalloprotease appears to function at the middle or late stage of fertilization. Ten *Tast* genes were identified in the *H. roretzi* genome database, among which four genes (*HrTast1*, *HrTast2b*, *HrTast2c*, and *HrTast3c*) possessed a single transmembrane domain in the N-terminal region. These four genes are transcribed in the testis and ovary, as revealed by RT-PCR. Anti-HrTast2c IgG raised against a peptide corresponding to the Zn-binding consensus sequence weakly inhibited fertilization at 0.5 mg/mL. These results led us to propose that sperm astacin-like metalloproteases may be involved in sperm penetration of the VC during *H. roretzi* fertilization.

## 1. Introduction

Fertilization is a key process in sexual reproduction and is essential for producing the next generation. In contrast to spermatozoa, eggs are surrounded by proteinaceous egg coats, called the vitelline coat (VC) or vitelline envelope in many marine invertebrates or the zona pellucida (ZP) in mammals, and by cellular coats, called follicle cells in ascidians or the cumulus oophorous in mammals [1,2,3,4,5]. These egg coats are responsible for protecting eggs and embryos from mechanical damage and for species-specific gamete interactions, which are particularly important in marine invertebrates. In deuterostomes, sperm protease(s) is believed to function as the lytic agent “lysin”, which generates a small hole for sperm penetration, because protease inhibitors inhibit the sperm penetration of the VC [6,7,8,9,10]. On the other hand, nonenzymatic lysin functions in protostomes, including shellfish, through species-specific binding and disrupting the interaction between filamentous proteins comprising the VC [7,11]. In contrast to those of protostomes, the molecular entity and functional mechanisms of lysin are poorly understood in deuterostomes [6,7,8,9,10].

In mammals, the sperm acrosomal trypsin-like protease acrosin [EC 3.4.21.10] has long been believed to be a zona-lysin because acrosin has potent proteolytic activity in the acrosome, a Golgi-derived small vesicle at the tip of the spermatozoa [12] (see also reviews [6,7,8]). However, in *acrosin*-KO mice, acrosin is not essential for fertilization or sperm penetration of the ZP, although a slight delay in the sperm penetration process of the ZP has been observed [13]. Rather, acrosin was proposed to play a role in the dispersal of the acrosomal contents [14]. Sperm proteases other than acrosin may play a cooperative role in fertilization in mice [15,16]. On the other hand, acrosin, which functions as a lysin, was recently revealed to be essential for fertilization in hamsters using *acrosin*-KO hamsters [17]. These results led us to consider that the function of acrosin depends on the species.

We have studied the sperm proteases involved in sperm penetration through the VC, which functions as a lysin, using the ascidian *Halocynthia roretzi* (Urochordata or Tunicata) because a large quantity of readily fertilizable sperm and eggs can be easily obtained from this species by controlling the seawater temperature and light conditions [18,19]. Hoshi et al. [18] first examined the effects of various protease inhibitors, including microbial inhibitors such as chymostatin (chymotrypsin inhibitor), leupeptin (trypsin inhibitor), antipain (papain and trypsin inhibitor), phosphoramidon (thermolysin [metalloprotease] inhibitor), and bestatin (aminopeptidase inhibitor); proteinaceous inhibitors such as soybean trypsin inhibitor (trypsin inhibitor), lima bean trypsin inhibitor, and potato protease inhibitor I (chymotrypsin inhibitor); and low-molecular-weight irreversible inhibitors such as phenylmethylsulfonyl fluoride (PMSF: serine protease inhibitor), tosyl-lysyl chloromethyl ketone (TLCK: trypsin inhibitor) and tosyl-phenylalanyl chloromethyl ketone (TPCK: chymotrypsin inhibitor), on fertilization. These experiments revealed that both trypsin inhibitors (leupeptin, antipain, and soybean trypsin inhibitors) and chymotrypsin inhibitors (chymostatin and potato proteinase inhibitor I) potently inhibited the fertilization of *H. roretzi.* Then, leupeptin and chymostatin were selected as representative inhibitors, and their effects on the fertilization of VC-deprived eggs were examined. Given that leupeptin and chymostatin showed no and weak inhibition of the fertilization of VC-free eggs, respectively, together with the proteinaceous inhibitor soybean trypsin inhibitor and potato proteinase inhibitor I inhibiting fertilization, they proposed that sperm surface trypsin-like protease(s) and chymotrypsin-like protease(s) play key roles in sperm penetration through the VC, most likely as lysins [18].

Sawada et al. purified two trypsin-like proteases, designated ascidian acrosin and spermosin, from sperm via the fluorogenic peptide substrate butoxycarbonyl (Boc)-Val-Pro-Arg-4-mehylcoumaryl-7-amide (MCA), which most potently inhibited fertilization among the peptidyl-Arg (or Lys)-MCA substrates [19] (see reviews [20,21]). By examining the inhibitory effects of various subsite-substituted peptidyl-argininals (leupeptin analogs) on fertilization and purified enzymes, both ascidian acrosin and spermosin were proposed to play key roles in fertilization [20,21]. However, these purified trypsin-like proteases have minimal or no ability to digest VC. Instead, sperm trypsin-like proteases and their precursors, particularly the C-terminal CUB domain of the ascidian proacrosin and the proline-rich region of the light chain of spermosin, may be capable of binding to the C-terminal region of vitellogenin, which is associated with the VC [20,21,22,23]. On the other hand, to identify chymotrypsin-like proteases involved in fertilization, Sawada and colleagues examined the effects of various peptidyl-Phe (or Tyr)-MCA substrates on fertilization and reported that succinyl (Suc)-Leu-Leu-Val-Tyr-MCA had the strongest inhibitory effect on fertilization [20,21]. The Suc-Leu-Leu-Val-Tyr-MCA-hydrolyzing protease was purified from *H. roretzi* sperm and identified as the proteasome [20,21]. Furthermore, the proteasome was released from sperm to the surrounding seawater upon sperm reaction [24], which is characterized by Ca^2+^ influx-mediated vigorous sperm movement and eventual shedding of a mitochondrion, which occurs on the VC during fertilization and is mimicked by the treatment of sperm with alkaline seawater [25]. In addition, when the supernatant of the sperm reaction was subjected to gel filtration, the proteasome antigen was eluted at a position of approximately 1000 kDa, the fraction of which exhibited VC-digesting activity [24]. Furthermore, we reported that the purified preparation of the sperm 26S-like proteasome degraded VC70, a 70 kDa VC component involved in self-sterility, in an ATP/ubiquitin (Ub)-dependent fashion [20,21,26], suggesting the extracellular function of the proteasome as VC lysin.

Sawada et al. reported that the proteasome may also be involved in the fertilization of *C. intestinalis* type A (Phlebobranchia) (another name: *Ciona robusta* [27]), which is useful for molecular biological studies because of the existence of a genome database [28,29,30]. To identify the sperm proteasome subunit on the surface of *C. intestinalis* sperm, proteomic analysis was subsequently performed via liquid chromatography-mass spectrometry (LC-MS) [31]. Although the proteasome subunits have not yet been identified, probably because of the limited amount on the sperm surface, considerable amounts of several peptides of astacin-like metalloproteases with thrombospondin type-1 repeats have been identified [32]. The sequences of these peptides revealed that five astacin-like metalloproteases with two or three thrombospondin type-1 repeats are located on the surface of the sperm of *C. intestinalis* type A [32]. To investigate the participation of these proteases in fertilization, the effects of metalloprotease inhibitors on fertilization were examined. Since hydroxamate inhibitors, including GM6001 and its analogs, are specific and potent inhibitors of metalloproteases compared with metal chelators such as EDTA, we utilized these inhibitors [31]. The results showed that the metalloprotease inhibitor GM6001 strongly inhibited the fertilization of intact eggs but not VC-deprived eggs [32]. In addition, after incubation of the isolated VC with intact sperm, several VC components, including CiVC57, a homolog of HrVC70, were degraded, and such degradation activity was inhibited by treatment with GM6001. These results suggest that the tunicate astacin-like metalloprotease with thrombospondin type-1 repeats (Tasts) plays a key role in the sperm penetration of the VC.

To investigate the involvement of metalloproteases in the fertilization of *H. roretzi*, we examined the effects of GM6001 and other metalloprotease inhibitors on the fertilization of *H. roretzi.* We report that GM6001-susceptible metalloproteases are involved in fertilization, most likely in sperm penetration through the VC in *H. roretzi.* Analogous to that in *C. intestinalis*, we propose that the functional homolog of Tast expressed in the testis may be a candidate protease(s) involved in this process.

## 2. Materials and Methods

### 2.1. Animals and Gametes

The ascidian *Halocynthia roretzi* (Type C), which had been aquacultured for human consumption in Onagawa Bay, Japan, was purchased from a fisherman and stored in the aquarium of the Research Center for Marine Biology, Graduate School of Life Sciences, Tohoku University. The sperm and eggs were collected as described previously [18,19]. Briefly, spawning was induced by controlling the seawater temperature at 13 °C and the light conditions during the breeding season. Mature eggs and sperm released from the gonoducts of the dissected gonads in a Petri dish filled with seawater were collected by pipetting them individually.

### 2.2. Fertilization Experiments

GM6001 and GM6001NC were purchased from EMD Millipore/Carbiochem/Merck (Chicago, IL, USA). TAPI-0, TAPI-1, and TAPI-2 were obtained from Peptide International (Louisville, KY, USA). MG115 was purchased from Peptide Institute (Minoh, Japan). The effects of metalloprotease inhibitors (GM6001, TAPI-0, TAPI-1, and TAPI-2) on the fertilization of *H. roretzi* were evaluated according to methods described previously [18,19]. Briefly, inhibitors that had been dissolved in DMSO at 10 mM were diluted with artificial seawater (ASW: 462 mM NaCl, 9.4 mM KCl, 10.8 mM CaCl_2_, 48.3 mM MgCl_2_, 10 mM EPPS (pH of 8.2)) [33]. A small volume (10 μL) of diluted nonself-sperm suspension was added to ASW containing the respective inhibitors and preincubated for 10 min at 13 °C. After preincubation, 20 μL of egg suspension (approximately 200 eggs) was gently mixed and incubated for 1 h at 13 °C, resulting in a total volume of 500 μL in 48-well multiwell dishes. The fertilization ratio was determined on the basis of the expansion of the perivitelline space at 1 h after insemination as described previously [18,19]. VC-free eggs were prepared according to methods described previously [32]. The fertilization rate of the VC-free eggs was determined on the basis of the first egg cleavage. Analyses on the effects of the timing of the addition of GM6001 or MG115 (a proteasome inhibitor, which is capable of inhibiting *H. roretzi* fertilization at 100 μM [34]) after nonself-sperm addition during fertilization were performed as described previously [35]. After insemination, a small volume (5 μL) of 10 mM GM6001 or MG115 was added to the egg suspension in ASW in a final volume of 500 μL.

### 2.3. RNA Preparation from Various Tissues/Organs and Reverse Transcription (RT)

The testis, ovary, gill, muscle, and hepatopancreas were collected from the sexually mature adult ascidian *Halocynthia roretzi* (Type C), which was obtained during the spawning season. One hundred milligrams of the respective tissues/organs were homogenized in 1 mL of RNAzol^®^RT according to the manufacturer’s protocol. Each homogenate was diluted with 0.4 mL of 0.1% diethylpyrocarbonate (DEPC)-treated water, incubated for 15 min, and then subjected to centrifugation (12,000× *g*, 15 min). To the resulting supernatant (1 mL), 0.4 mL of 75% ethanol was added, followed by incubation for 10 min. The insoluble RNA was collected by centrifugation (12,000× *g*, 12 min), and the pellet was washed twice with 75% ethanol, followed by centrifugation (8000× *g*, 3 min). The pellet was dissolved in 50 μL of DEPC-treated water and used for total RNA preparation (3 μg). Reverse transcription was performed to obtain cDNA via the SuperScript III First-Strand Synthesis System for RT-PCP (Invitrogen (Waltham, MA, USA)) according to the manufacturer’s protocol. As a negative control (-RT), DEPC-treated water was used instead of reverse transcriptase.

The obtained cDNAs were used as templates for PCR of the astacin-like metalloprotease genes. The forward primers for the respective metalloproteases were synthesized on the basis of the sequences at a region in the first exon, whereas the reverse primers were prepared on the basis of the sequences in the third exons (for primer sequences, see Table 1). PCR was performed using these primer pairs. PCR for EF1α was also performed via the primers listed in Table 1 as a reference for the respective cDNA preparations. The PCR products were subjected to agarose gel electrophoresis and visualized with ethidium bromide. Furthermore, these PCR products were ligated into the T-Vector pMD20 (TaKaRa (Kyoto, Japan)), and the plasmids from randomly selected positive colonies were sequenced.

### 2.4. BLAST Search of Tast-Homologous Gene Models in H. roretzi

*H. roretzi* homologs to CiTast were searched via BLAST via the ascidian genome database [30] (ANISEED: Ascidian Network for In Situ Expression and Embryological Data) (https://aniseed.fr (accessed on 17 November 2024)), and the CiTast1 sequence (MH108630) was used as a query. The presence of the transmembrane domain was explored via DeepTMHMM (https://services.healthtech.dtu.dk/service.php?DeepTMHMM (accessed on 17 November 2024)). The signal peptide was searched via SignalP-5.0 (https://services.healthtech.dtu.dk/services/SignalP-5.0/ (accessed on 17 November 2024)).

### 2.5. Preparation of Antibodies Against HrTast2c and Their Effects on Fertilization

Two peptides corresponding to the sequence of the HrTast2c Zn-binding consensus sequence (position 1: CAIIQHEMLHLLGFAHE (residues 238–254)) and the unique sequence (position 2: CGGGSKWTDWGEWSA (residues 371–384)) were synthesized, conjugated to keyhole limpet hemocyanin and subjected to immunization of rabbits, which was carried out by the Sigma-Aldrich Japan (Tokyo, Japan). The titer was checked via ELISA (for details, see Figure S3). Immunoglobulin G (IgG) was obtained from the respective antisera via protein A–agarose chromatography. For the fertilization experiments, the sperm suspension was preincubated for 10 min with IgG preparations, which had been dialyzed against ASW, before the fertilization experiments.

### 2.6. Sperm Extraction

A portion (0.2 g) of frozen *H. roretzi* sperm was thawed, suspended in 1.2 mL of 1% Triton X-100 in 50 mM Tris/HCl (pH of 8.0) and homogenized, followed by centrifugation at 9000× *g* for 20 min. The supernatant (sperm extract) was subjected to SDS-PAGE and Western blotting as described previously [22].

## 3. Results

### 3.1. Effects of Metalloprotease Inhibitors on Fertilization

**Figure 1:**
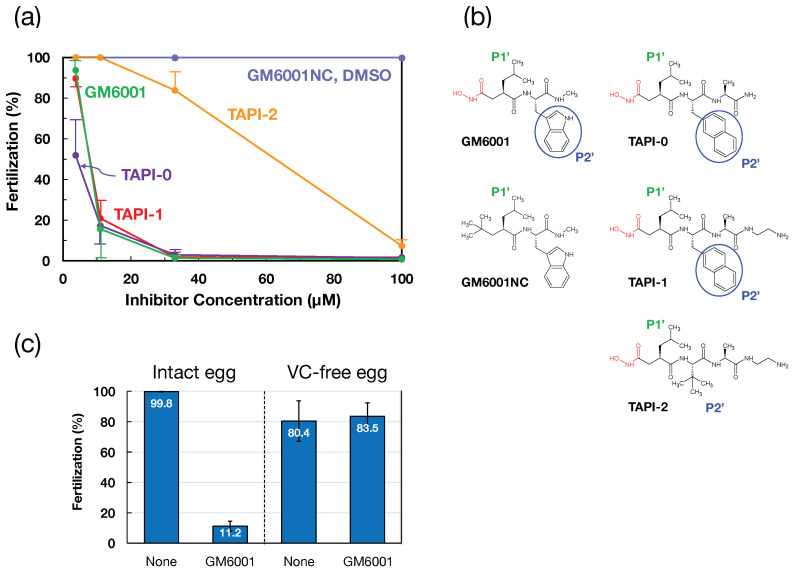
Effects of metalloprotease inhibitors on *H. roretzi* fertilization. (**a**) Metalloprotease inhibitors (TAPI-0 (purple), TAPI-1 (red), GM6001 (green), and TAPI-2 (orange)) were dissolved in DMSO at a concentration of 10 mM and were serially diluted 3-fold with ASW. The final inhibitor concentrations are indicated on the abscissa. GM6001NC (negative control; blue) is an analog of GM6001 without a metal-chelating hydroxamate group. The final concentration of DMSO (blue) used as a vehicle was 1% at 100 μM. Error bar: SE (*n* = 5). (**b**) The structures of the compounds used in (**a**) are illustrated. Every compound has a Leu residue at the P1′ position (green), whereas only strong inhibitors such as GM6001, TAPI-0, and TAPI-1 possess an aromatic ring at the P2′ position (blue circle). Notably, MG6001NC, lacking a hydroxamate group, has no inhibitory activity, even with the presence of an aromatic ring at the P2′ position (not indicated by a blue circle). For the nomenclature of the P1′ and P2′ positions, see [37,38]. (**c**) Effects of GM6001 on the fertilization of intact and VC-free eggs of *H. roretzi*. None, 0.1% DMSO; GM6001, 25 μM GM6001 containing 0.1% DMSO. Error bar: SE (*n* = 3).

The effects of metalloprotease inhibitors (GM6001, TAPI-0, TAPI-1, and TAPI-2) on *H. roretzi* fertilization were examined. As depicted in Figure 1a, TAPI-0, TAPI-1, and GM6001 strongly inhibited fertilization at 33 and 11 μM, whereas TAPI-2 weakly inhibited fertilization at 33 μM. On the other hand, DMSO had no inhibitory effect at a concentration of 1%, representing the concentration used with the 100 μM inhibitor. As shown in Figure 1b, the P2′ positions of GM6001, TAPI-0, and TAPI-1 have an aromatic ring, suggesting the importance of an aromatic ring at the P2′ position for the strong interaction between the enzyme and inhibitor (see Figure 1b; for the reactive site and nomenclature of the P2′ subsite, see [36,37,38]). GM6001NC (negative control of GM6001), which lacks the reactive-site hydroxamate group, showed no inhibition of fertilization, even with the presence of a P2′ aromatic ring (Figure 1a,b).

**Figure 2:**
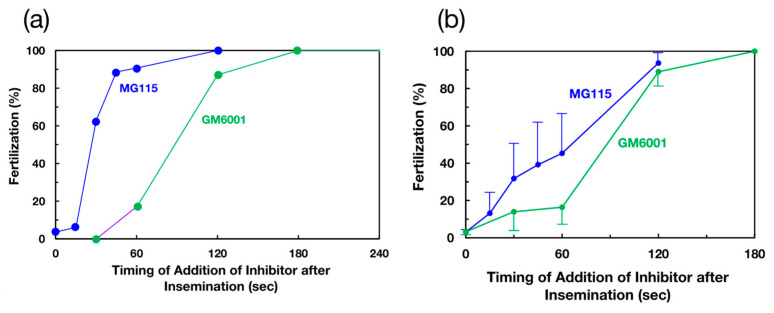
Effects of the timing of the addition of GM6001 (metalloprotease inhibitor; green) or MG115 (proteasome inhibitor; blue) after nonself-sperm addition during fertilization. After insemination, a small volume (5 μL) of 10 mM GM6001 or MG115 was added to the egg suspension in ASW in a final volume of 500 μL. After incubation at 13 °C for 1 h, the fertilization ratio was determined by counting the number of total eggs and fertilized eggs on the basis of VC elevation. (**a**) One representative result. (**b**) Five experimental results (mean ± SE).

This potent inhibitory effect of GM6001 did not affect the fertilization of VC-free eggs (Figure 1c), suggesting that a metalloprotease is responsible for sperm penetration through the VC. To investigate the timing of the participation of a metalloprotease during fertilization, the effect of the addition of GM6001 after insemination on fertilization was examined and compared with the effect of the addition of MG115 (a proteasome inhibitor). One representative result and the combined results of five experiments are depicted in Figure 2a,b, respectively. GM6001 strongly inhibited fertilization even 1 min after insemination. The inhibitory ability was greatly reduced 2 min after insemination. In contrast, the inhibitory potency of the proteasome inhibitor MG115 diminished 30 s after the addition of sperm. Although both timings appear to be similar in Figure 2b, the timings of both inhibitors were always distinctly different (see Figure 2a). These results support our previous assumption that the sperm proteasome functions at the early stage of fertilization [21,35]. Although our present results cannot exclude the possible involvement of the metalloprotease in the binding of sperm to the VC, the metalloprotease obviously functions at the middle or late stage during the sperm passage of the VC. Compared with acrosin [20,35], which functions between 2 and 5 min after insemination, the metalloprotease appears to participate in fertilization at an earlier timepoint than does acrosin.

### 3.2. Candidate Astacin-like Metalloproteases Involved in Fertilization

**Figure 3:**
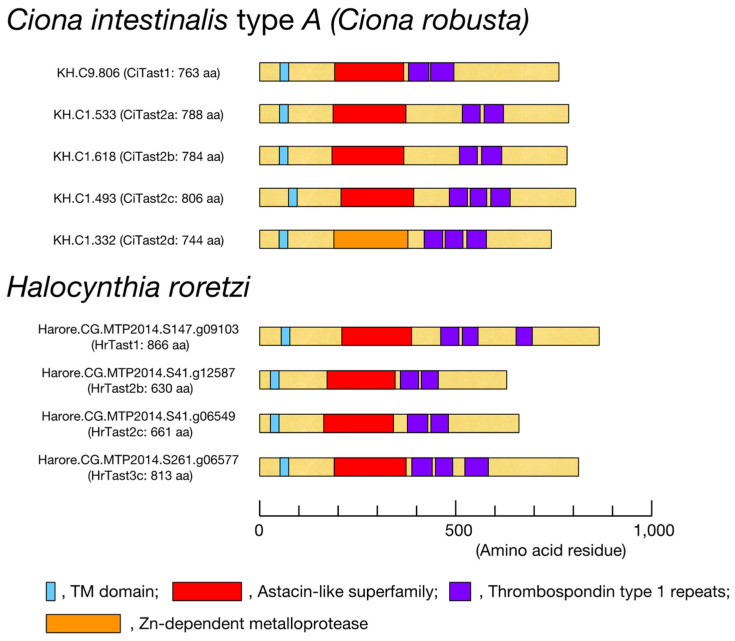
Schematic drawings of four *H. roretzi* Tast (astacin-like metalloprotease with thrombospondin type 1 repeats) proteins and domains, as predicted from the respective gene models. The domain organization, including the transmembrane domain, of *Ciona intestinalis* type A Tast proteins (CiTast1, CiTast2a, CiTast2b, CiTast2c, and CiTast2d) is also illustrated in the upper panel.

In *C. intestinalis*, five Tast proteins (Tast1, Tast2a, Tast2b, Tast2c, and Tast2d) have been identified on the sperm surface via LC-MS analysis [31,32]. However, no structural evidence exists that these proteins are membrane proteins or are exposed to the extracellular surface. We then reassessed them for the presence of a transmembrane domain and signal peptide via DeepTMHMM and SignalP-5.0, respectively. We observed that all five Tast proteins have no signal sequence but possess a single transmembrane domain in the N-terminal region, and the C-terminal region is predicted to be outside, i.e., they are type II transmembrane proteins (Figure 3). Although two other gene models are present in *C. intestinalis* that possess an astacin-like metalloprotease domain and a thrombospondin type-1 repeat, these putative proteins have no transmembrane domain. These findings support our previous conclusion that CiTast1, CiTast2a, CiTast2b, CiTast2c, and CiTast2d are located on the sperm surface [31,32]. These findings led us to consider that metalloproteases expressed in the testis that possess a transmembrane domain, an astacin-like metalloprotease domain, and a thrombospondin type-1 repeat may be candidate sperm metalloproteases involved in *H. roretzi* fertilization.

**Figure 4:**
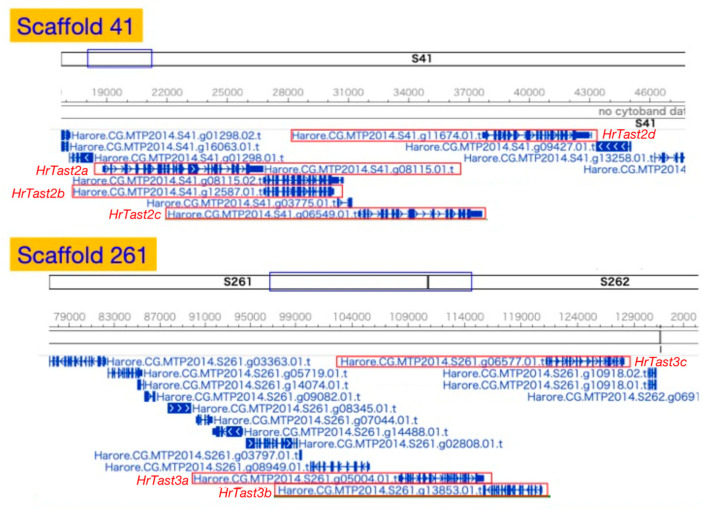
Genomic organization of clustered *H. roretzi Tast* genes in scaffold 41, where *HrTast2a*, *2b*, *2c*, and *2d* are contiguously located, and in scaffold 261, where *HrTast3a*, *3b*, and *3c* contiguously reside.

**Figure 5:**
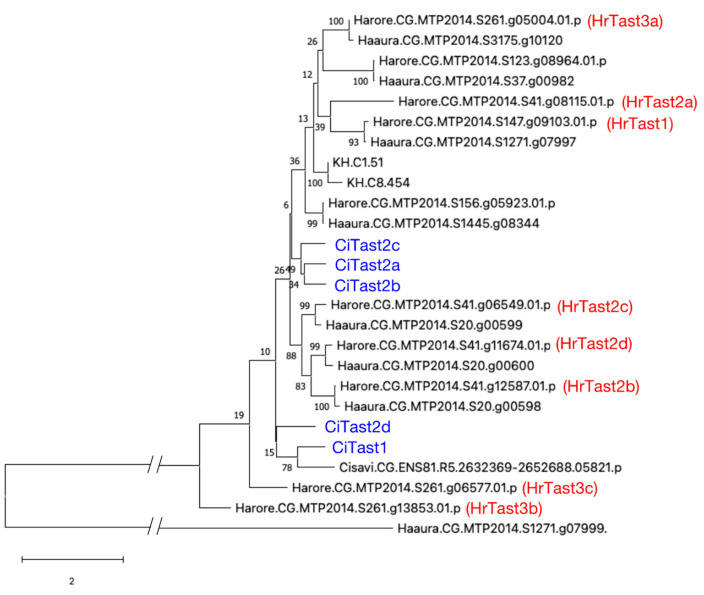
Molecular phylogenetic tree of astacin-like metalloproteases with thrombospondin type-1 repeats. Proteins containing an astacin-like metalloprotease domain and thrombospondin type-1 repeat were multialigned via MEGAX and viewed via a phylogenetic tree. *Ciona intestinalis* type A (indicated by CiTast or KH), *Ciona savignyi* (Cisavi), *Halocynthia roretzi* (Harore or HrTast), and *Halocynthia aurantium* (Haaura). To clarify the molecular phylogenetic relationship, CiTast1, 2a, 2b, 2c, and 2d were indicated by blue, while HrTast1, 2a, 2b, 2c, 2d, 3a, 3b, and 3c were indicated by red.

In this context, a BLAST search of the *H. roretzi* gene model was performed using the *C. intestinalis* Tast1 sequence as a query and ANISEED as an ascidian genome database. Ten gene models possessing an astacin-like metalloprotease domain and two or more thrombospondin type-1 repeats were identified (Table 2). Among them, four genes, designated *HrTast2a*, *HrTast2b*, *HrTast2c*, and *HrTast2d*, were tandemly located in this order in scaffold 41 (Figure 4). On the other hand, three genes, *HrTast3a*, *HrTast3b*, and *HrTast3c*, also resided contiguously in scaffold 261 in this order (Figure 4). Molecular phylogenetic analysis revealed that a close relationship did not exist between CiTast and HrTast, although orthologs were observed between Tast proteins from *H. roretzi* and *H. aurentium* (Figure 5).

Among the 10 gene models of *HrTast*, only four HrTast proteins, HrTast1, HrTast2b, HrTast2c, and HrTast3c, contained a single transmembrane domain (Table 2, Figure 3). These four proteins are predicted to be type II transmembrane proteins, namely, the astacin-like domain and thrombospondin type-1 repeat are located outside. These results are not inconsistent with our assumption that these metalloproteases located on the surface of sperm cells function extracellularly during fertilization, probably as VC lysin.

Schematic drawings of the total size and domain organization of the four HrTast proteins are depicted in Figure 3. All four gene products contained a single transmembrane domain, followed by an astacin-like metalloprotease domain and two or three thrombospondin type-1 repeats, which are predicted to be in the extracellular region. These features are very similar to those of CiTast proteins, albeit with no orthologous relationship (see Figure 5).

### 3.3. Transcription of HrTast Genes in H. roretzi

**Figure 6:**
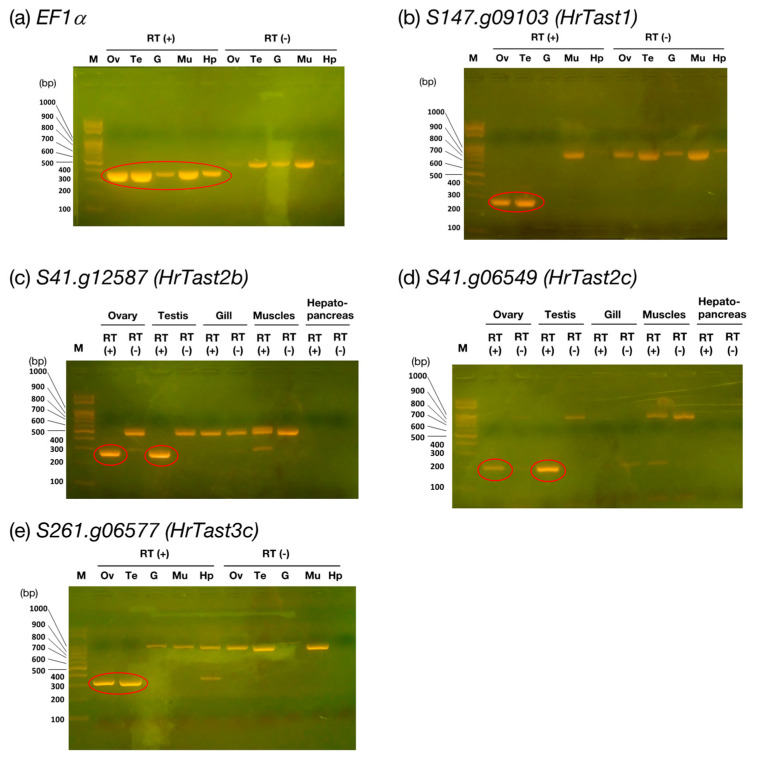
mRNA expression of *HrTast* genes. The ovary, testis, gill tissue, muscle tissue, and hepatopancreas were dissected from each sexually mature *H. roretzi* during the spawning season, from which RNAs were isolated and subjected to RT-PCR using the respective primers indicated in Table 1. The PCR products were subjected to agarose gel electrophoresis and visualized via UV irradiation of gels stained with ethidium bromide. The PCR products from cDNA are denoted with red circles. The *EF1a* gene was used as an internal standard and is expressed in every tissue or organ. M, marker; Ov, ovary: Te, testis: G, gill; Mu, muscle; Hp, hepatopancreas.

The transcription of the above four genes was investigated via RT-PCR with RNAs obtained from the ovary, testis, gill, muscle, and hepatopancreas of *H. roretzi* as a template, and the primers used are listed in Table 1. As shown in Figure 6, all four genes tested were transcribed in the testis and ovary but not in the other tissues or organs, whereas the EF1*α* gene was transcribed in every tissue. The band size was identical to the size expected from the respective gene model except for HrTast1. However, the sizes of the four PCR products coincided well with those of the determined sequences (Figure S2). These results indicate that four genes (HrTast1, HrTast2b, HrTast2c, and HrTast3c) are transcribed in the testis.

The nucleotide sequences of the PCR products were determined (see Figure S2). The numbers of nucleotides are 275 bp (*HrTast1*), 287 bp (*HrTast2b*), 200 bp (*HrTast2c*), and 334 bp (*HrTast3c*). The accession numbers of the sequences of the PCR products of *HrTast1*, *HrTast2b*, *HrTast2c*, and *HrTast3c* are LC849074, LC849104, LC849102, and LC849103, respectively.

### 3.4. Effects of HrTast2c Antibodies on Fertilization

**Figure 7:**
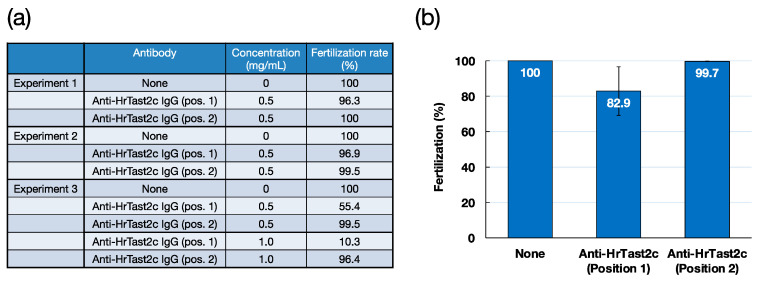
Effects of anti-HrTast2c peptide antibodies (positions 1 and 2) on fertilization. Two peptide antibodies against the HrTast2c Zn-binding consensus sequence (position 1) and the unique sequence (position 2) were generated in rabbits. The IgG preparations, which were obtained from the respective antisera as described in the Materials and Methods, were dialyzed against ASW and preincubated with sperm at a final concentration of 0.5 mg/mL. After preincubation, a small volume of egg suspension was mixed in a total volume of 500 μL. Three independent fertilization experiments were carried out, and the results are listed in (**a**). The combined data at 0.5 mg/mL are depicted in (**b**). Error bar, SE (*n* = 3).

To investigate the role of HrTast in fertilization, two peptides corresponding to HrTast2c position 1 (Zn-binding site) and position 2 (unique sequence in HrTast2c with high antigenicity) were synthesized, and peptide antibodies were obtained as described in the Materials and Methods. Both antibodies showed high reactivities to antigenic peptides on the basis of ELISA (Figure S3). However, in the Western blotting of the sperm extract, the position 1 antibody produced no appreciable band, whereas the position 2 antibody produced several specific bands with molecular masses in the range of 50–100 kDa, which were blocked by the antigenic peptide (see Figure S3). Both antibodies were not useful for immunocytochemistry. Interestingly, the position 1 antibody, but not the position 2 antibody, weakly inhibited fertilization at a concentration of 0.5 mg/mL (Figure 7). The inhibitory ability appears to be variable among individuals. However, since the position 2 antibody (IgG) did not inhibit fertilization in different batches, the inhibitory effects on the fertilization of the position 1 antibody (IgG), which reacts to the Zn-binding consensus sequence, suggest the possible participation of HrTast2c in fertilization. Further studies are necessary to investigate the localization and roles of four HrTast metalloproteases, HrTast1, HrTast2b, HrTast2c, and HrTast3c, in fertilization.

## 4. Discussion

In the present report, we demonstrated the participation of metalloproteases in the fertilization of the Stolidobranch ascidian *Halocynthia roretzi.* The metalloprotease inhibitor GM6001 inhibited the fertilization of intact eggs but not VC-free eggs; therefore, metalloproteases susceptible to GM6001 appear to be responsible for sperm penetration of the VC. The timing experiment of GM6001, which was added after insemination, also revealed that the metalloproteases in question must be involved in sperm penetration of the VC. We also identified four candidate genes (*HrTast1*, *HrTast2b*, *HrTast2c*, and *HrTast3c*) expressed in the testis that possess an astacin-like metalloprotease domain and two or three thrombospondin type-1 repeats after a single transmembrane domain. Among these four metalloproteases, at least HrTast2c may be involved in *H. roretzi* fertilization since the antibody against the metal-binding site inhibited fertilization.

We previously reported that five astacin-like metalloproteases (CiTast1, CiTast2a, CiTast2b, CiTast2c, and CiTast2d) on the sperm surface play key roles in sperm penetration through the VC in the Phlebobranch ascidian *C. intestinalis* type A [32]. These five gene products were confirmed to be localized on the surface of sperm cells via LC-MS analysis [31,32]. We previously attempted to disrupt the *CiTast* genes via genome editing. However, the targeted embryos delayed their hatching and elicited abnormal metamorphosis. Similar results were obtained by adding GM6001, suggesting that the phenotype is not due to off-target effects. Most likely, metalloproteases are involved in the sperm penetration of the VC, hatching, and metamorphosis. Therefore, obtaining *Tast*-gene-knockout adult individuals is nearly impossible. Although GM6001 can inhibit many metalloproteases, only Tast was detected among the metalloproteases on the surface of *C. intestinalis* sperm, which led us to conclude that these Tast proteins play a key role in sperm penetration of the VC. VC components, including CiVC57, are promising candidates for the natural substrates of CiTast given that CiVC57 and other bands newly appeared after incubation with intact sperm inhibited with GM6001 [32]. For *Halocynthia roretzi*, we carried out a similar preliminary experiment: the isolated VC was incubated with the sperm extract and subjected to SDS-PAGE (Figure S4). Although a 120 kDa band was newly detected after incubation of the VC with the sperm extract, we failed to identify the 120 kDa protein via LC-MS analysis (Table S1). Although 62 protease-digested fragments of vitellogenin (453.5 kDa), covering 22% of the entire region, were identified, no convincing evidence was obtained that supports the idea that “120 kDa proteins” are mixtures of the fragments of vitellogenin located on the VC (Figure S5) [22,23]. Further studies are necessary to explore the substrate for HrTasts.

The results of the timing of inhibitor addition indicated that the metalloprotease functions at the middle or late stage during fertilization. We previously reported the effects of the timing of inhibitor addition during fertilization in *H. roretzi* [35]. On the basis of these results, we propose that chymotrypsin-like protease, which was later identified as the proteasome [20,21,24], and spermosin, a sperm trypsin-like protease with narrow substrate specificity, function at the early stage between 1 and 3 min [20,21,35], whereas acrosin, a sperm trypsin-like protease with broad substrate specificity, functions at the very late stage, between 2 and 5 min after insemination [21,35]. Compared with the proteasome and acrosin, the metalloprotease appears to function later than the proteasome but earlier than acrosin. Therefore, we cannot exclude the possibility that the sperm proteasome may be involved in the activation of the sperm metalloprotease. In any case, our present results indicated that the proteasome and metalloprotease are both involved in fertilization, probably as a VC lysin or its proteolytic activator.

Tast proteins from *H. roretzi* and *H. aurantium* are closely related, and these *Tast* genes seem to be orthologs. Therefore, *Tast* genes are likely produced and duplicated in some regions before speciation to the corresponding species. On the other hand, a close relationship was not detected between Tast proteins from *Ciona intestinalis* type A and *Halocynthia roretzi* or between those from *Ciona intestinalis* type A and *Ciona savignyi*. In *C. intestinalis* type B, we could not identify a gene with an astacin-like metalloprotease domain and a thrombospondin type-1 repeat in the ascidian genome database. However, these results do not necessarily deny the possibility of the presence of orthologs since whether the genome databases of *C. savignyi* and *C. intestinalis* type B cover all the genes is unclear.

**Figure 8:**
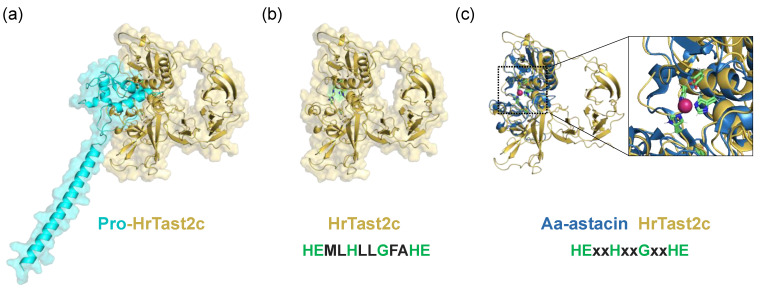
Prediction of the 3D structure of HrTast2c with AlphaFold2. (**a**) Full-length HrTast2c (inactive pro-HrTast2c) containing the pro-domain (cyan). (**b**) Active HrTast2c. The Zn-binding segments (green) in the active site are exposed after the pro-domain is removed. (**c**) Superposition of Aa-astacin (blue, PDB ID: 1AST [36]) and AlphaFold2-predicted HrTast2c (yellow). Zn-binding segments are shown as green sticks, and Zn^2+^ ions are shown in red. The Zn-binding segments are highly conserved between these two proteins. The inset shows a zoomed-in view of the catalytic center of the protease domain.

Astacin family members are characterized by the consensus sequence HEXXHXXGXXHE, which is the Zn-binding domain [36]. Generally, to be an active form of metalloprotease, cleavage of the N-terminal pro-domain is necessary. To the best of our knowledge, the occurrence of type II transmembrane-type metalloproteases has not been reported [39,40,41]. Most transmembrane-containing metalloproteases, such as MMPs and ADAM, have a transmembrane domain at the C-terminal region [39]. In the case of type II transmembrane proteins, the N-terminal region is located in the intracellular region and may be unable to inactivate the active site of the metalloprotease. To address this issue, we predicted the 3D structure of HrTast2c with AlphaFold2 (Figure 8). The results showed that the active site is occupied by the N-terminal pro-domain (Figure 8a) even if the N-terminal transmembrane domain (residues 21–43) is embedded in the sperm membrane (Figure 3 and Table 2). On the other hand, the active site of HrTast2c is predicted to be exposed if the N-terminal pro-domain is removed (Figure 8b) at the processing site (between Gly-49 and Ala-50) of *Astacus astacus* Aa-astacin [36] (for sequence alignment of HrTast2c and Aa-astcin, see Figure S3). The 3D structure of Aa-astacin was superimposed on the 3D structure of HrTast2c, suggesting that the active sites of HrTast2c and Aa-astacin are highly conserved (Figure 8c). Since the active site of HrTast2c is protected by the pro-domain, the antibody against the active site of HrTast2c may not easily access the active site of HrTast2c. This may explain why the position 1 antibody could not strongly inhibit fertilization. Further detailed studies are necessary to clarify the activation mechanisms of Tasts on a structural basis.

The mammalian homolog of Tast, which has an astacin-like metalloprotease domain and two or three thrombospondin type-1 repeats, is not known. However, interestingly, the acrosome of mouse sperm contains acrosin and MMP2 as the main proteases [42]. These proteases are thought to be involved in sperm penetration of the ZP [42]. Moreover, a proteasome inhibitor (MG132), metalloprotease inhibitor (o-phenanthroline), and serine protease (acrosin) inhibitor (benzamidine) inhibited the degradation of recombinant human ZP4, ZP2, and ZPs, respectively, using capacitated human sperm [43]. In line with our observations, mammalian sperm may also utilize metalloprotease(s) for sperm penetration of the ZP in addition to the proteasome, although the sperm proteasome is believed to be a zona-lysin [9,10]. We cannot exclude the possibility of the cooperative participation of metalloproteases in the sperm penetration process of the ZP. Further studies are necessary to elucidate whether metalloproteases, in addition to the proteasome, are involved in sperm penetration of the ZP in mammals.

## Figures and Tables

**Table 1:** Primers used for RT-PCR.

Primer Name	Sequence (5′ -> 3′)
S147.g09103 (HrTast1) Forward	CTAGGAAACCGCACCATCAT
S147.g09103 (HrTast1) Reverse	GCACGTGTTGATCATGTAAGG
S41.g12587 (HrTast2b) Forward	CACAAACGACGATCACCTTC
S41.g12587 (HrTast2b) Reverse	ATTCATTTTTCCGGCATCAC
S41.g06549 (HrTast2c) Forward	GGCTCTTGGATTCATCATCG
S41.g06549 (HrTast2c) Reverse	CGTGTGCACAGGTAGTGACG
S261.g06577 (HrTast3c) Forward	AAGAACCCAAAGCACGTGAG
S261.g06577 (HrTast3c) Reverse	CGCTTTCTTGGGAAGTTTCA
HrEF1α Forward	GGGAAGAGTGGAGACTGGA
HrEF1α Reverse	CTTACCAGAGCGACGATCG

**Table 2:** Tast gene models of metalloproteases in *H. roretzi*.

Gene Model *	Name	Total aa	Domain **
S147.g09103	HrTast1	866	TM, ZnMc_astacin-like, TSP1, TSP1, TSP1
S41.g08115	HrTast2a	987	ZnMc, TSP1, TSP1, TSP1, TSP1, TSP1, TSP1
S41.g12587	HrTast2b	630	TM, ZnMc_astacin-like, TSP1, TSP1
S41.g06549	HrTast2c	661	TM, ZnMc_astacin-like, TSP1, TSP1
S41.g11674	HrTast2d	733	ZnMc_astacin-like, TSP1, TSP1, TSP1
S261.g05004	HrTast3a	1002	ZnMc_astacin-like, TSP1, TSP1, TSP1, TSP1, TSP1
S261.g13853	HrTast3b	778	ZnMc, TSP1, TSP1, TSP1
S261.g06577	HrTast3c	813	TM, ZnMc_astacin-like, TSP1, TSP1, TSP1
S156.g05923	HrTast4	533	SP, ZnMc_astacin-like, TSP1, TSP1
S123.g08964	HrTast5	566	ZnMc_astacin-like, TSP1

* “Harore.CG. MTP2014” is abbreviated prior to the scaffold number (S). ** The following abbreviations are used for domains: TM, transmembrane domain (red letter); ZnMc_astacin-like, Zn-dependent metalloprotease, astacin-like protease; ZnMC, Zn-dependent metalloprotease; TSP1, thrombospondin type 1; SP, signal peptide.

## Data Availability

The original contributions presented in the study are included in the article/supplementary material, further inquiries can be directed to the corresponding author.

## Supplementary Materials

The following supporting information can be downloaded at: https://www.mdpi.com/article/10.3390/biom14121487/s1, Figure S1: Nucleotide and predicted amino acid sequences of the HrTast1, HrTast2b, HrTast2c and HrTast3c gene models (website: https://www.aniseed.fr/ (accessed on 17 November 2024)); Figure S2: Determined nucleotide sequences (upper) of the RT-PCR products of the HrTast1, HrTast2b, HrTast2c and HrTast3c genes and the predicted nucleotide sequence (lower) from the respective gene model; Figure S3: Preparation and Western blotting of HrTast2c peptide antibodies; Figure S4: SDS-PAGE of the VC incubated with sperm extract; Figure S5: Vitellogenin fragments were detected in the 120-kDa band via SDS-PAGE; Table S1: LC-MS analysis of the 120-kDa protein, which newly appeared after incubation with the isolated VC and sperm extracts.
