# Supplementary material for: Involvement of Metalloproteases in the Fertilization of the Ascidian Halocynthia roretzi
Source: Biomolecules. 2024 Nov 22;14(12):1487. doi: 10.3390/biom14121487 (PMC11673157; doi:10.3390/biom14121487)
Supplement: Supplementary file 1 [file biomolecules-14-01487-s001.zip › biomolecules-3187679-supplementary.pdf]

Involvement of Metalloproteases in the Fertilization of the Ascidian *Halocynthia roretzi*

Hitoshi Sawada [1,2] *, Ikuya Hattori [1,2], Noritaka Hashii [3] and Takako Saito [4]

1 Sugashima Marine Biological Laboratory, Graduate School of Science, Nagoya University, 429-63 Sugashima, Toba, Mie 517-0004, Japan
2 Department of Food and Nutritional Environment, College of Human Life and Environment, Kinjo Gakuin University, Omori, Moriyama-ku, Nagoya, Aichi 463-8521, Japan
3 Division of Biological Chemistry and Biologicals, National Institute of Health Sciences, 3-25-26 Tonomachi, Kawasaki, Kanagawa 210-9501, Japan
4 Department of Applied Life Sciences, Faculty of Agriculture, Shizuoka University, Shizuoka 422-8529, Japan

* Correspondence: hsawada1954@gmail.com

Supplemental Data

Figure S1. Nucleotide and predicted amino acid sequences of the HrTast1, HrTast2b, HrTast2c and HrTast3c gene models (website: https://www.aniseed.fr/)

Figure S2. Determined nucleotide sequences (upper) of the RT–PCR products of the *HrTast1, HrTast2b, HrTast2c* and *HrTast3c* genes and the predicted nucleotide sequence (lower) from the respective gene model.

Figure S3. Preparation and Western blotting of HrTast2c peptide antibodies

Figure S4. SDS-PAGE of the VC incubated with sperm extract

Figure S5. Vitellogenin fragments were detected in the 120-kDa band via SDS–PAGE

Table S1. LC–MS analysis of the 120-kDa protein, which newly appeared after incubation with the isolated VC and sperm extracts *

Predicted amino acid sequence of HrEF1a

>Harore.CG.MTP2014.S31.g10104.01.p

MVKEKFHINIVVIGHVDSGKSTTTGHMIYKCGGIDKRTIDKFEKEAQETGKGSFKYAWVLDKLKAERERG
ITIDIALWKFETTKYYITIIDAPGHRDFIKNMITGTSQADCAVLIIAAGTGEFEAGISKSGQTREHALLA
FTLGVKQMICAINKMDNTEPPYSEARFKEIVTEVSSYMKKVGYNPKKIPFVPISGWHGDNMMEKSDKTPW
YKGFTKMSDKPVGATLKEAFDLIEQPTRPTDKPLRLPLQDVYKIGGKCIGTVPVGRVETGIIKPGQIVKF
SPVNLTTEVKSVEMHHESLVEGCPGDNVGFNVKNVSVKDIRRGNVCGDAKNCPPQKTSSFLAQVIILNHP
GNISKGYTPVLDCHTAHIACKFAEIKEKIDRRSGKTMEENPKSVKSGDAAMIELIPSKPMCVEKFSEFPP
LGRFAVRDMRQTVAVGVIKDVTVAVETGGKVTKAAMKSKGKK

cDNA sequence of HrEF1a model

(PCR product using the primers in Table 1 (highlighted sequences): 364 nucleotides)

>Harore.CG.MTP2014.S31.g10104.01.t

TCCACCTCATCGTTCCTTTTAGCGTGCGGCATCTTGGTCTTGAGTTCCAATTCTTTGTCAAAAATAAGAA
AACACCATGGTTAAAGAAAAATTTCATATTAACATCGTCGTAATTGGTCATGTCGACTCCGGCAAGTCCA
CTACTACTGGCCATATGATATATAAATGTGGGGGTATTGACAAAAGAACAATCGACAAGTTTGAAAAAGA
AGCTCAAGAGACGGGTAAAGGTTCGTTCAAGTATGCCTGGGTGTTGGATAAGTTGAAGGCTGAGAGAGAA
CGTGGTATTACCATCGACATTGCTCTATGGAAGTTTGAAACCACCAAATACTATATCACCATCATTGATG
CCCCTGGACATCGTGATTTCATTAAAAACATGATCACAGGAACATCTCAAGCAGACTGTGCTGTCCTCAT
TATAGCGGCTGGTACTGGGGAATTCGAAGCTGGAATTTCTAAGAGTGGCCAAACGAGAGAGCATGCTCTT
TTGGCTTTCACCCTTGGTGTGAAACAGATGATTTGTGCTATTAACAAAATGGACAACACGGAGCCTCCAT
ACAGCGAAGCTAGATTCAAGGAAATCGTCACGGAAGTCTCTTCCTACATGAAGAAAGTTGGCTACAATCC
GAAAAAAATTCCATTCGTGCCCATCTCTGGATGGCATGGTGATAACATGATGGAAAAGTCTGACAAAACC
CCTTGGTATAAAGGATTCACGAAAATGAGTGACAAGCCAGTTGGTGCTACTCTAAAAGAAGCCTTTGATC
TTATTGAGCAACCAACACGTCCCACTGATAAACCCTTGCGTTTGCCTCTACAAGATGTGTACAAAATAGG
AGGCAAGTGTATTGGAACAGTGCCAGTGGGAAGAGTGGAGACTGGAATTATAAAACCAGGCCAGATTGTT
AAGTTTTCTCCTGTAAATCTTACGACTGAAGTCAAATCTGTCGAAATGCATCATGAGAGTTTAGTGGAGG
GCTGTCCCGGAGATAACGTCGGTTTTAACGTCAAGAACGTGTCTGTCAAAGACATTCGCAGAGGTAATGT
GTGTGGGGATGCGAAGAATTGCCCTCCACAGAAAACTAGTAGTTTCTTAGCTCAGGTTATAATCTTGAAC
CATCCCGGAAACATTTCAAAGGGCTACACGCCAGTTTTGGACTGCCATACTGCTCACATCGCTTGCAAGT
TCGCAGAGATCAAGGAGAAAATCGATCGTCGCTCTGGTAAGACTATGGAGGAAAACCCAAAGAGTGTAAA
GTCTGGTGATGCTGCCATGATTGAGCTAATTCCCTCGAAACCAATGTGTGTTGAAAAGTTTTCCGAATTC
CCTCCTCTGGGTCGTTTTGCTGTCCGTGACATGCGTCAGACGGTTGCAGTCGGTGTTATCAAGGATGTTA
CGGTTGCTGTCGAAACGGGAGGAAAAGTGACAAAAGCCGCCATGAAAAGTAAAGGCAAGAAGTGACTCGA
ACCGGAGAATGATCGCTCTCGAACTAATTTAGGATTAAAAAGCTCTTTTGTGTTAATATTGGTGTTAATT
TTGTCATGTCGCATGTTTCACTTGGTCATATTTTTGAACTCTACTCACCGTAAACATTTCCGGCGGAAAA
AACCTGTCTTTTACTAGCTGACTGGCGTGGTTGTCCCCTAAGATTTCATGTGATAAATGTAAATTGTATG
AATCAATAAATTTC

Predicted amino acid sequence of HrTast1

>Harore.CG.MTP2014.S147.g09103.01.p aa（Molecular mass: 99.43 kDa）

RLFHSTLQQQLSRMPHPPKPRKPHHHRRTKRTGVGIRRAYQPLTECQTLFKKISAPQKKKISPSANKVPW
MFASVVVSFLAAIAVVIGVIVAKSQKSLLEQKCEELLKRINSNETDTSISPYMINTCIEVGSDCNFEYTS
CQSPCPFGYARDKESGCYTCACAQEGLHEMDIQFHEEDVAGLLEDYGYKLLFNDEEESFSLFISSVKKSV
GLWTKWQKNGRYKVPYEIDAKLTSGYSDVIRESIQVISKESCIDLIPRDKEKDYIVFISLRGCWAKVGYR
TWIQDVSIGLGCDKVEIVIHEILHALGFYHEHSRPDRDGFVDVLLENVKEDMKNNFKKLIEGETQDLNSE
YDIKSVMHYDGLSFTKNFKPTMTYKGTQDYVDISGILTLSSEDKDELNLLYKCKPENIGQTVTGSWSEWG
SWNDCYMTCKDHETPYRRKFRKCVDKDGNGVVGCEGKTSIREECPVPYCEKAGAEWEHWGAWSECSKSCG
KGKRNRERKCPYSGECSMFYGGYFDRDSCATHKCGEKPELKWTLWTVWLACKGECGEGVQKRIRKCEHGD
ELVEDCSGGKYEEDETGIGYETQSCVVECTTPLVSLSSEPPTTPETIAMTSATTTAGPNKTPTKTTTKT
PNTIAKTTTKATGPTTTTPVTTNRATTEKITETANPDWRAWGLWGSCSKSCDGGQRERERVCKSYTCEGD
KKMSEYCNTRSCRAQSRPHTIFSATEAIIPIFCPYGRIVPGDFNADRRTDLLCLFSDGYIRIYLANARGV
FDEVGWEGQTNFCQFSEDSLAPPQVFAKDLNADKFIDILCYNKRSGKMQILLNQKLQFHTKVDWEGKKMN
CDVNMGAVIQLQDVNGDSRADLVCGDEKTRVKEIFLNKT

cDNA sequence of HrTast1 model

(PCR product using the primers in Table 1 (highlighted sequences): 314 nucleotides)

>Harore.CG.MTP2014.S147.g09103.01.t nt

AGGTTATTTCATTCCACGCTGCAGCAACAACTTAGCAGG**ATG**CCTCATCCTCCAAAACCTAGGAAACCGC
ACCATCATCGTCGTACCAAGAGGACTGGAGTTGGAATTAGAAGAGCATATCAACCTTTAACTGAATGCCA

AACGCTTTTTAAAAAAATTTCAGCACCTCAGAAAAAAAAAATATCACCATCAGCTAATAAAGTGCCATGG
ATGTTTGCATCTGTTGTTGTCAGCTTTCTTGCAGCCATAGCAGTCGTCATCGGGGTAATAGTAGCCAAAT
CTCAAAAGAGTCTACTGGAACAGAAATGTGAAGAACTTTTGAAAAGAATAAATTCAAACGAAACAGACAC
ATCTATTTCTCCTTACATGATCAACACGTGCATCGAAGTGGGCAGTGACTGTAACTTTGAATACACCTCT
TGTCAGTCTCCGTGTCCTTTTGGATATGCCAGAGATAAAGAATCGGGATGCTACACATGTGCTTGTGCAC
AAGAAGGACTTCATGAAATGGACATTCAGTTCCATGAAGAAGATGTGGCTGGATTACTGGAGGATTACGG
TTACAAACTTCTTTTCAATGACGAAGAAGAATCTTTTTCTTTGTTTATATCTTCAGTTAAGAAAAGTGTT
GGGTTGTGGACAAAATGGCAGAAAAATGGAAGATACAAAGTCCCTTATGAAATAGATGCAAAATTAACCT
CTGGATATTCAGATGTCATTCGTGAAAGCATTCAAGTCATTTCAAAAGAAAGCTGTATCGATTTGATACC
GAGAGACAAAGAGAAGGACTATATTGTCTTTATTTCTCTCAGAGGCTGTTGGGCAAAAGTAGGGTACAGG
ACATGGATCCAAGATGTTTCTATCGGCTTGGGTTGCGACAAGGTTGAAATCGTTATTCATGAGATCCTGC
ATGCTCTTGGCTTTTATCACGAGCACTCAAGACCAGACAGAGATGGATTTGTGGATGTGCTCCTTGAAAA
TGTCAAAGAAGATATGAAAAACAACTTCAAGAAACTGATTGAAGGGGAAACTCAAGATTTAAACTCGGAG
TATGACATCAAATCGGTGATGCATTACGACGGTTTGTCATTTACTAAAAATTTCAAGCCGACAATGACAT
ACAAAGGAACACAAGATTATGTCGACATCTCTGGTATATTAACACTTTCTTCTGAAGACAAAGACGAATT
GAACCTGCTGTACAAATGCAAGCCTGAGAATATTGGGCAAACTGTCACAGGTTCATGGTCTGAATGGGGC
AGCTGGAACGACTGCTACATGACGTGCAAGGATCACGAAACGCCCTACAGAAGAAAATTTAGAAAGTGTG
TCGACAAGGATGGGAACGGTGTTGTCGGTTGCGAAGGAAAAACTTCCATCAGGGAGGAATGTCCGGTTCC
ATATTGCGAGAAAGCTGGAGCGGAGTGGGAACATTGGGGAGCGTGGTCAGAATGTAGTAAGTCCTGTGGC
AAAGGAAAACGAAACAGGGAACGAAAATGTCCTTATTCCGGAGAATGTAGTATGTTCTATGGCGGATACT
TTGATCGTGACTCCTGTGCTACTCATAAATGTGGTGAAAAACCCGAACTAAAATGGACTCTTTGGACAGT
CTGGTTGGCTTGCAAGGGAGAATGTGGTGAAGGTGTACAAAAGAGAATAAGGAAATGTGAACATGGTGAC
GAACTAGTAGAAGATTGTTCTGGTGGAAAGTATGAGGAAGATGAAACTGGTATTGGGTACGAGACGCAGT
CATGCGTCGTTGAATGCACTACTCCTCTTGTTTCATTGTCAAGTGAACCTCCAACAACTCCTGAAACAAT
CGCAATGACCAGCGCTACCACGACAACCGCTGGACCAAACAAAACTCCCACAAAAACCACAACGAAGACA
CCAAACACGATTGCCAAAACCACCACAAAAGCCACAGGACCGACCACAACCACTCCTGTTACCACTAACA
GGGCAACTACTGAAAAAATAACAGAAACTGCAAATCCAGATTGGAGAGCATGGGGTTTGTGGGGATCTTG
CTCAAAATCTTGCGACGGCGGCCAAAGAGAAAGAGAAAGAGTTTGCAAAAGTTATACTTGTGAAGGAGAC
AAGAAAATGTCAGAATATTGTAACACGCGCTCTTGCAGAGCACAAAGTCGACCGCACACAATATTCTCAG
CTACGGAAGCCATCATTCCAATATTTTGTCCTTATGGGAGAATCGTGCCCGGCGATTTCAACGCTGATCG
TAGAACTGATTTGCTCTGCCTGTTCTCTGATGGTTACATTCGTATATACCTGGCGAACGCTCGCGGAGTG
TTTGATGAGGTAGGCTGGGAAGGTCAAACCAACTTCTGTCAATTCTCTGAAGATTCTTTAGCACCTCCCC
AAGTCTTCGCTAAAGATCTTAATGCAGATAAATTCATTGATATCTTGTGCTACAATAAGCGCTCGGGGAA
AATGCAGATTTTGTTGAACCAGAAATTACAGTTCCATACAAAAGTTGATTGGGAGGGAAAGAAAATGAAC
TGTGATGTGAACATGGGCGCAGTTATACAGCTGCAGGATGTAAATGGTGACAGTAGAGCTGATCTTGTTT
GTGGAGATGAAAAAACGAGAGTCAAGGAAATATTTCTGAATAAGACTTAGTACGGAACATAATAAGTTTA
TCACGAAGCTATTTACTTGAGTTCAGCTTCAATCAAGTCTGTTTTGTTGTGATTTAAAAAAAAAATACTT
GGTCAGCCTCATTTAGTACTGATTCGAACAGTTTTTCCTATTAATGGAACTGGTCCATATTTCTAAATA
AGACTTAATACGGAATATATTAAGTTTATCACAAAGCTAGTTACTTGAGTTCAGCTTCAATCAAGTCTGT
TTTGTTGTGATTTAAAAAAAAAATACTTGGTCAGCCTCATTTAGTACTGATTCGAATAGTTT

Predicted amino acid sequence of HrTast2b
>Harore.CG.MTP2014.S41.g12587.01.p （Molecular mass: 70.24 kDa）
MESKDPPGPSTVKGRSMTVRRKNVYIMFGVVVFTAIGIITVVSILLQGSTRMISELRYYSKMSCDAGKMN
SPIQINECMKATCIFDPQACISPCPFGYRKNKRGCDVSCACASEGNFQGDVHFDEDSLPELSELYGHTER
AGDFSVFASSVESSTKLWNNNLVDGRHKIPYQMDSLLGKAAKGAIKEALSTFTTKSCIDFIPWTDEKHSV
LFKAEAGCWSEIGKNMKKSPQVLSVGDSCEKTGIVQHEVLHMLGFQHEHVRPDRDAYVIVDYDNIIATNV
IQFNKLEQTEVVDLGSEYDFSSLLQFSSDMFSKNGKDTITNRVTGGEILGQQIEFSVGDIRELNSLYNCY
EDKPGKWTEWGQWSLCDQTCGQGRKRRYRSCLDGSDSVKGGNLEYLECSETPCPSKDGGWGKWGEYDECS
NSCGGGYQWRLRECSQDDCEGSESEGKKCNLQSCFDRSLSDETSQTEGKPKPGPDFFENTEVTIPLNLCD
VKTYNEWYFGDFNGDKRTDTLCINPDGEAKLAYTNDRGILTEPQWEGKLEGCLNCIKYVGDFNGDGKDDV
LCKNIYSRTIAIKFTTSDGIFDEGFTDAGNFCGNDFHVVIVSDVNGDGKADIICGSRDTNIEIRINRFEK

cDNA sequence of HrTast2b model
(PCR product using the primers in Table 1 (highlighted sequences): 287 nucleotides)
>Harore.CG.MTP2014.S41.g12587.01.t
AGTCTGTTATCTAGCATATCACAAACGACGATCACCTTCTCTTTCCTGTGAAGAATAAGACTCCAATCCT
TTGTTATTGGAAAGCCTCGCGTCATC**ATG**GAATCCAAGGATCCTCCCGGTCCATCAACTGTTAAAGGAAG
ATCCATGACCGTGAGAAGGAAAAATGTATACATTATGTTTGGTGTTGTCGTCTTTACAGCAATTGGGATC
ATTACTGTAGTTTCTATCTTGTTGCAAGGTAGCACAAGAATGATCTCTGAACTACGATACTATTCAAAAA
TGTCTTGTGATGCCGGAAAAATGAATTCACCAATTCAAATTAATGAATGCATGAAGGCTACATGTATATT

CGATCCGCAAGCTTGCATTTCTCCGTGTCCTTTTGGGTACAGAAAAAACAAGAGAGGATGCGATGTCAGT
TGTGCTTGTGCATCAGAAGGCAATTTTCAAGGAGATGTACATTTTGATGAAGATTCCCTTCCTGAATTGT
CTGAGTTGTATGGACACACAGAACGTGCTGGAGACTTTTCAGTGTTTGCTAGTTCTGTAGAATCGAGTAC
AAAGTTATGGAACAATAACCTTGTTGATGGCCGCCACAAAATTCCATATCAAATGGATTCTTTATTGGGG
AAAGCTGCGAAGGGTGCAATAAAAGAGGCGCTTAGCACATTTACAACGAAATCCTGCATCGATTTTATAC
CGTGGACAGACGAGAAACATTCGGTTTTATTCAAGGCTGAAGCCGGTTGCTGGTCTGAAATAGGAAAAAA
CATGAAAAAATCTCCCCAAGTCCTCTCAGTTGGAGATAGCTGTGAAAAAACAGGAATAGTTCAGCATGAA
GTGCTTCACATGTTGGGTTTCCAACACGAACACGTCCGGCCTGATCGAGACGCTTACGTGATTGTCGATT
ATGATAATATAATAGCAACCAACGTCATCCAATTTAACAAACTGGAACAAACCGAGGTTGTTGACTTGGG
TTCGGAATATGATTTCAGTTCGTTGCTGCAGTTCTCCAGCGATATGTTCAGTAAGAACGGTAAAGACACC
ATCACCAACCGTGTGACAGGTGGAGAGATTCTAGGACAACAGATTGAATTTTCTGTGGGAGACATACGAG
AACTTAACTCTCTTTATAATTGCTATGAGGATAAACCAGGAAAATGGACAGAATGGGGGCAATGGAGCTT
GTGTGACCAAACATGCGGACAAGGGAGAAAGAGGAGGTACAGGAGTTGTTTGGATGGAAGTGACAGTGTC
AAAGGAGGCAATCTGGAATACCTGGAGTGCTCAGAAACACCATGCCCTAGTAAAGACGGTGGCTGGGGGA
AGTGGGGTGAATACGACGAGTGTAGCAACTCATGTGGGGGAGGATATCAGTGGAGGTTGAGAGAGTGCTC
ACAAGACGATTGCGAAGGATCTGAAAGTGAAGGAAAGAAATGTAATCTACAATCCTGTTTTGATAGATCA
CTTTCTGACGAAACTTCACAGACGGAGGGCAAACCAAAACCAGGCCCCGATTTCTTCGAGAACACTGAGG
TCACCATCCCGCTAAATCTATGTGATGTCAAAACATATAACGAATGGTATTTCGGAGATTTTAACGGTGA
CAAACGAACCGACACTCTGTGCATAAATCCGGATGGAGAGGCAAAACTTGCCTATACAAATGACCGTGGA
ATTCTGACAGAGCCTCAGTGGGAAGGAAAGCTGGAGGGATGTTTAAATTGTATCAAGTATGTCGGAGACT
TCAATGGAGATGGTAAAGATGATGTTCTGTGCAAAAATATCTATTCGAGAACGATAGCCATCAAGTTCAC
TACCTCGGATGGAATTTTCGATGAAGGTTTTACAGATGCGGGAAACTTTTGCGGCAACGATTTTCACGTT
GTCATCGTCAGCGACGTGAATGGTGACGGAAAAGCCGACATTATTTGCGGCTCTCGAGATACCAATATTG
AAATAAGAATAAACCGATTCGAGAAATAGAATTTTAACATGATTTTCCGTGGATTTTATATAAGACTCTA
TTGACTTTGTAAAAATCTTAATTCTGTTCGGGCTCGGTAGCGTTCCTTTTTTATGCTTGGTTTTTTACTT
TA

Predicted amino acid sequence of HrTast2c
>Harore.CG.MTP2014.S41.g06549.01.p (Molecular mass: 72.84 kDa)
MNSNRKHRTRTQADSKKKLHLLALGFIIVLIAGILGVIITLVQGSAKKPIAEQRMYARMSCDDETPNSLE
VNECQKATCMFDVTTCAHDCPFGYQPDARGCDIGCDCATESNFQGDIHFDEHTLPTYMKMFRAKSEDDFE
IFSGSAVHAYPLFNENPQNGRHRIPYELDNSRGQLAVTQIEEAIKIYSEKTCIDFVPKTSSDGQDFVKFS
KGPGCWAFIGKTKYPQDVSIGVECGTTAIIQHEMLHLLGFAHEQCRPDRDGYVQIKKENINPRNLHNFDK
KTEDEISDLGSEYDVKSIMQYDSLSWSTNGQSTMVEKKTGAFIFAKHEFAESDLYELNKLYDCLDHVEGE
KGSEEGGEEGGEEGDEEGGGGGGSKWTDWGEWSACPRTCGPANLRRYRFCLDSSDQLVTGCEGYDQDASI
SCSGPTCPGIIAEWGSWGEYGDCTKTCGMGLKFRFRNCSVEGECWTGWAGEGEFCNPEACNYPASGHAGV
GGKDGGGQDSVSGVRYFKDDPLKIESSLCGKDASTDWLFGDVDGDKKIEAICIDSQGKTEIGRPDENGMA
MHTNWKGNMDGCSGRYRYTGDVDGDGKDDIICKDETAGMLKVKFAGEKGEFDSSFTGVGNFCTDEKQGML
LFDVNGDGKIDILCQSIDLSMEIRLNNFESN

cDNA sequence of HrTast2c model
(PCR product using the primers in Table 1 (highlighted sequences): 200 nucleotides)
>Harore.CG.MTP2014.S41.g06549.01.t
AGGTTAACACCTTTATAGCTTGTTATAAAAATTTGCTTAGGGTTGAATTCCATCAGATGCAAGTGATTAT
ATGAAGTATTAGACGTTGTTGATC**ATG**AATTCGAACAGAAAGCATAGAACCAGAACCCAAGCGGATTCAA
AGAAGAAACTTCATCTGTTGGCTCTTGGATTCATCATCGTTCTTATAGCTGGCATTCTTGGTGTAATCAT
AACTCTTGTACAAGGCAGCGCTAAAAAACCTATAGCAGAACAAAGAATGTATGCAAGAATGTCATGTGAT
GATGAAACGCCAAATTCATTGGAAGTCAATGAATGCCAAAAGGCAACTTGCATGTTTGACGTCACTACCT
GTGCACACGACTGTCCGTTTGGTTACCAACCAGACGCTAGGGGGTGTGATATTGGCTGTGACTGTGCCAC
GGAAAGCAATTTTCAAGGGGATATTCATTTTGATGAACATACACTTCCTACATATATGAAGATGTTCAGG
GCAAAGTCAGAAGATGACTTTGAAATATTTTCGGGGTCAGCTGTGCATGCATACCCTTTATTCAATGAAA
ATCCTCAGAATGGAAGACATAGAATCCCTTACGAACTAGACAACAGTAGAGGTCAATTAGCTGTAACTCA
AATTGAAGAAGCAATCAAGATATATTCAGAAAAAACTTGCATCGATTTTGTTCCAAAGACTAGCAGTGAC
GGACAGGACTTTGTTAAATTTTCTAAAGGCCCTGGATGTTGGGCATTCATTGGAAAGACAAAATATCCCC
AAGACGTCTCAATTGGAGTTGAATGTGGAACAACGGCAATCATACAACATGAAATGCTTCACTTGCTAGG
ATTTGCACACGAGCAGTGTAGACCTGATAGAGATGGTTATGTTCAAATCAAGAAGGAAAATATTAACCCA
AGAAATTTGCACAACTTTGATAAAAAGACGGAAGACGAAATTTCGGACCTAGGTTCAGAATACGATGTAA
AATCCATAATGCAGTACGACAGCCTATCCTGGAGCACAAACGGTCAAAGTACAATGGTAGAAAAAAAAAC
AGGAGCATTCATATTTGCAAAACACGAATTTGCTGAATCGGACCTGTATGAACTTAATAAATTGTATGAT
TGCCTGGACCACGTAGAGGGTGAAAAAGGTAGTGAAGAAGGTGGTGAGGAAGGTGGTGAAGAAGGTGATG
AAGAAGGTGGAGGTGGAGGAGGATCAAAGTGGACCGATTGGGGAGAATGGAGTGCCTGTCCAAGGACGTG

CGGGCCTGCGAATTTGAGGAGATATAGATTTTGTCTGGATTCATCAGATCAGCTGGTCACCGGCTGCGAA
GGTTATGACCAGGATGCATCCATTTCTTGCAGTGGACCCACTTGTCCCGGAATAATCGCGGAGTGGGGTA
GCTGGGGAGAATATGGCGACTGTACTAAGACTTGCGGCATGGGACTCAAGTTCAGATTCAGGAATTGCAG
TGTCGAAGGGGAGTGTTGGACTGGTTGGGCTGGAGAAGGAGAATTTTGCAATCCAGAGGCGTGTAACTAT
CCTGCAAGTGGTCATGCTGGTGTGGGTGGTAAGGATGGTGGTGGTCAAGATTCAGTTTCAGGGGTGAGAT
ATTTCAAGGATGATCCGCTGAAAATTGAATCATCTCTGTGCGGAAAAGATGCAAGCACCGATTGGCTTTT
CGGGGATGTCGATGGGGACAAAAAAATAGAAGCTATTTGCATCGATAGCCAAGGAAAAACAGAGATAGGA
CGTCCAGACGAAAATGGGATGGCAATGCACACAAACTGGAAGGGAAACATGGATGGCTGCTCTGGTAGGT
ACAGATATACTGGAGATGTCGATGGAGACGGAAAGGATGATATCATTTGCAAAGATGAAACTGCTGGAAT
GTTAAAAGTAAAATTTGCCGGAGAGAAAGGAGAATTCGATTCTTCTTTCACGGGTGTTGGAAATTTCTGC
ACCGATGAAAAGCAGGGCATGTTACTGTTTGATGTGAACGGCGACGGAAAAATCGACATTCTTTGTCAAT
CTATCGATCTATCTATGGAAATCAGACTGAACAATTTTGAATCCAATTGAATTTAATGATGTATATGTGT
ATGAACAAAAACTATTTTGATTCAGTATACGGCTCGTGAGCCAGCCGCAAGGCTACTCACGTTTGAAGCT
TGAACTCAAATTTGTTGTGTATAGTTCTTATTAAGTCTGTCCATCTCTGATATATATACTAAAATGGCTT
TAATTTTTAGTGTTTGTTACAATTTGTTAATTTGGCATATTATTTTTGTAGACTGGATACACAGCGTTAC
TTAGTCCATATCATTTTCATTCCATATACAGTCTGTTTGAATTCTGTTAACAGTCTTTAATTCCTGAGTC
AGGATTTGGCAACTTAAAGAATCTAATTGTTAATAGTGTTGTCTTTAGGGAAGAAAATGCTCTTAGCCTT
ATGGATAAAGGACTAATAATCTATGTTTTAAGTATAATTCATTATTTTTCCTTGTCGCATGTTACTAGTT
AATGCAGTCAAAAGGTAAGCCATATAGCAGAAACTTACATAATAAATTACTGGGCCCTGGTGAGAAATAA
TGACTCCTACTTTTTACAATGTTGTTCAATACTTACTGTTCCTGTATTTATTTCTACAGA

Predicted amino acid sequence of HrTast3c

>Harore.CG.MTP2014.S261.g06577.01.p（Molecular mass: 91.18 kDa）

MESSKKQKRSERSADRPRSKNPKHVSAKAGDRSSARDKKKPAQNSSNKVLLSIVVCVVLAAAIGALMFAL
FGMSTSDCQKYLKSQAKGGKNKDIALSPIAISECLETMCTFDVLQCNAPCPFGFETSQESGCTKSCDCAV
EGKFQGDVHFGADDVPEMLEKFGHKEEDEYEVFFATTKMLWNNLEMEGRFKVPYKMMPGLPKEDYQVWVD
AIGNLSKLICIDLIPWSDEGDFLEIVPIGSSCSAFIGRHHGRNQVNLANGCFFQTTIHHELFHTFGFFHE
HSRPDRDGHVNIYFDNIWPQKKHNFDKEKKENIKDLDTEYDFQSAMQYPSYIFAEEKTKPTITGKPGDII
RGNQIRHHYLTPMDVLELKKLYECPEDEAKAKEGEWGDWLKWSDCSDTCGGGLMSRFRGCFHKPTGTQLT
IGCEGMPVEYKACNEDECPALTGKFLEWKEWSKCGKTCGEGHHSRVRHCSEVLQCTGPYFDYKPCIVALC
DTGEREIFSNILEHQLSEIQAVVDGIYGEWADWFEWEHCDVTCGTGERLRLRICKRKEKEIATNRCKADE
KIGKVYRDGQAQVEECKIRDCKDVPPGLTETKSPTQGPPPTIPTKNDASSGNTPSPPGPAPVNTPSSSGP
TPAPINTPSKRPTPSKDKPKVTGIFKEENSQVKLLCMYGLLLSGKFNSDKYEDLMCLYPDGYMYVYYSKG
EKQSGAVTGKISWHGPTSLCAFVVYKINYKIYARDMDDDGDDDIVCYNPLTREVEIGINNGGKFRAVKDW
RGQRQSCDSVIHIEDVNGDGKNDLVCGNADNAVKQVYLNGYGD

cDNA sequence of HrTast3c model

(PCR product using the primers in Table 1 (highlighted sequences): 334 nucleotides)

>Harore.CG.MTP2014.S261.g06577.01.t

TTTATGAAATCTGAACTTGATTATACAGGTTTTGCAATTAGGATATTTTCATAATATTGTCTCTTGCCAA
TCGTGATCGTGCT**ATG**GAATCTAGCAAGAAACAAAAGAGATCTGAAAGAAGCGCAGACCGGCCTCGAAGT
AAGAACCCAAAGCACGTGAGCGCGAAAGCCGGGGATCGCTCTTCAGCTCGAGATAAGAAGAAGCCCGCAC
AAAACTCATCCAACAAAGTTTTGCTCAGCATTGTAGTTTGCGTTGTTCTTGCTGCGGCAATCGGAGCTTT
GATGTTTGCTTTATTTGGAATGTCAACATCGGATTGTCAAAAGTATCTGAAATCTCAAGCTAAAGGCGGC
AAAAATAAAGATATTGCGTTAAGTCCTATTGCGATCTCAGAATGTTTAGAAACCATGTGTACATTTGACG
TTCTTCAATGCAACGCCCCATGCCCTTTCGGCTTTGAAACTTCCCAAGAAAGCGGATGTACAAAAAGTTG
CGACTGCGCTGTCGAAGGAAAATTTCAGGGGGATGTTCATTTTGGTGCGGATGATGTCCCGGAAATGTTG
GAGAAATTCGGACACAAAGAAGAAGATGAATATGAGGTCTTTTTTGCAACCACCAAGATGCTTTGGAACA
ATCTAGAGATGGAAGGACGATTTAAAGTCCCCTACAAAATGATGCCTGGACTACCTAAAGAAGATTATCA
AGTATGGGTGGATGCAATAGGAAATTTATCGAAGCTAATTTGCATAGATTTGATACCGTGGTCGGACGAA
GGGGATTTTCTTGAAATCGTTCCCATAGGGAGTTCATGTAGTGCGTTTATTGGCAGGCATCATGGACGTA
ATCAAGTAAATCTTGCTAATGGTTGTTTTTTTCAAACCACCATTCACCATGAACTATTCCATACATTCGG
ATTCTTTCACGAGCATTCACGACCAGACCGTGATGGACATGTCAATATCTATTTTGACAATATTTGGCCG
CAAAAAAAGCACAATTTTGATAAGGAAAAAAAAGAAAATATTAAGGATCTTGACACTGAATATGACTTTC
AATCAGCCATGCAGTATCCGAGTTATATCTTCGCTGAGGAAAAAACAAAGCCGACTATCACGGGTAAGCC
TGGTGACATCATACGTGGTAACCAGATTAGGCATCATTATCTCACCCCCATGGATGTCTTGGAGCTAAAA
AAGTTGTATGAATGTCCTGAAGATGAGGCAAAGGCAAAAGAAGGTGAGTGGGGTGATTGGTTGAAGTGGA
GTGATTGCAGCGATACTTGCGGAGGTGGACTCATGAGTCGTTTCAGAGGTTGTTTTCATAAACCAACCGG
AACTCAGTTAACTATTGGATGCGAAGGCATGCCTGTCGAATATAAAGCATGCAATGAAGACGAGTGCCCT
GCTCTAACTGGCAAATTTTTGGAATGGAAGGAATGGTCTAAGTGTGGAAAAACATGTGGTGAAGGCCATC
ATTCTAGAGTTAGGCATTGCAGTGAAGTTCTTCAATGTACAGGTCCTTACTTCGACTACAAGCCATGCAT

```
TGTCGCACTATGTGACACTGGGGAACGTGAGATATTTTCAAATATACTAGAACATCAATTATCTGAGATA
CAGGCTGTAGTAGATGGGATATATGGGGAATGGGCTGATTGGTTCGAATGGGAACATTGTGATGTTACTT
GTGGAACGGGAGAAAGGCTAAGACTTAGGATATGTAAAAGAAAGGAAAAAGAAATAGCCACAAATCGATG
CAAAGCAGATGAAAAAATAGGGAAGGTTTATAGAGACGGACAGGCCCAAGTCGAGGAATGTAAAATTCGA
GACTGTAAAGATGTGCCCCCTGGACTTACTGAAACCAAATCACCCACCCAGGGTCCACCACCTACCATAC
CGACGAAAAATGATGCGAGCTCTGGAAATACACCTTCACCACCTGGACCAGCCCCCGTGAATACTCCATC
CTCATCAGGACCCACTCCTGCTCCCATAAACACCCCATCCAAGCGACCTACCCCAAGTAAAGATAAACCC
AAAGTAACAGGTATTTTCAAAGAAGAAAACTCCCAGGTTAAATTGTTGTGTATGTATGGTCTTTTGCTAT
CTGGGAAGTTCAACTCAGATAAGTATGAAGATTTGATGTGCCTATATCCAGATGGCTACATGTATGTGTA
TTATTCGAAAGGTGAAAAACAAAGTGGCGCTGTGACAGGAAAAATCAGCTGGCATGGTCCTACTTCTTTG
TGCGCTTTTGTAGTCTACAAGATTAACTATAAGATATACGCAAGAGATATGGATGACGACGGCGATGATG
ATATTGTGTGCTACAATCCGCTTACGCGGGAAGTTGAAATTGGGATTAACAATGGCGGTAAGTTCCGTGC
TGTGAAGGACTGGAGAGGACAACGACAGTCATGCGATAGTGTCATACACATTGAAGATGTGAATGGCGAT
GGAAAAAACGATTTGGTTTGTGGAAATGCTGACAATGCCGTTAAACAAGTCTATCTCAATGGCTATGGTG
ATTAGCAACAGAACTAAAAGTGATTTGGATATATCAGAAGGTAATATAAATCTGCGTCTTGCATCGATGC
AAAAATATATTGCCATTATTCTACTTTCAAAAAAACTTGCATTTGTTTATAAAACTTGATTTACATTCAT
GT
```

Figure S1. Nucleotide and predicted amino acid sequences of the HrTast1, HrTast2b, HrTast2c and HrTast3c gene models (website: https://www.aniseed.fr/)

The yellow and green highlighting indicate the forward and reverse primer sites, respectively. The cDNA sequences highlighted in yellow, purple and green indicate the predicted PCT products. The nucleotide sequence underlined in HrTast1 was missing in RT-PCR, suggesting the presence of an intron sequence. The initiation methionine codon (ATG) is indicated by a bold letter.

HrTast1: Determined nucleotide sequence of RT-PCR product (275 nt)

CTAGGAAACCGCACCATCATCGTCGTACCAAGAGGACTGGAGTTGGAATTAGAAGAGCACCTCAGAAAAAAAAAATAT
CACCATCAGCTAATAAAGTGCCATGGATGTTTGCATCTGTTGTTGTCAGCTTTCTTGCAGCCATAGCAGTCGTCATCG
GGGTAATAGTAGCCAAATCTCAAAAGAGTCTACTGGAACAGAAATGTGAAGAACTTTTGAAAAGAATAAATTCAAACG
AAACAGACACATCTATTTCTCCTTACATGATCAACACGTGC

HrTast1: Predicted nucleotide sequence from the gene model (Gray-highlighted sequence was lacking in the cloned cDNA, suggsting this region is intron.)

CTAGGAAACCGCACCATCATCGTCGTACCAAGAGGACTGGAGTTGGAATTAGAAGAGCATATCAACCTTTAACTGAAT
GCCAAACGCTTTTTAAAAAAATTTCAGCACCTCAGAAAAAAAAAATATCACCATCAGCTAATAAAGTGCCATGGATGT
TTGCATCTGTTGTTGTCAGCTTTCTTGCAGCCATAGCAGTCGTCATCGGGGTAATAGTAGCCAAATCTCAAAAGAGTC
TACTGGAACAGAAATGTGAAGAACTTTTGAAAAGAATAAATTCAAACGAAACAGACACATCTATTTCTCCTTACATGA
TCAACACGTGC

HrTast2b: Determined nucleotide sequence of RT-PCR product (287 nt)

CACAAACGACGATCACCTTCTCTTTCCTGTGAAGAATAAGACTCCAATCCTTTGTTATTGGAAAGCCTCGCGTCATCA
TGGAATCCAAGGATCCTCCCGGTCCATCAACTGTTAAAGGAAGATCCATGACCGTGAGAAGGAAAAATGTATACATTA
TGTTTGGTGTTGTCGTTTTTACAGCAATTGGGATCATTACTGTAGTTTCTATCTTGTTGCAAGGTAGCACAAGAATGA
TCTCTGAACTACGATACTATTCAAAAATGTCTTGTGATGCCGGAAAAATGAAT

HrTast2b: Predicted nucleotide sequence from the gene model (Identical sequence between the predicted sequence and determined sequence except one nucleotide)

CACAAACGACGATCACCTTCTCTTTCCTGTGAAGAATAAGACTCCAATCCTTTGTTATTGGAAAGCCTCGCGTCATCA
TGGAATCCAAGGATCCTCCCGGTCCATCAACTGTTAAAGGAAGATCCATGACCGTGAGAAGGAAAAATGTATACATTA
TGTTTGGTGTTGTCGTCTTTACAGCAATTGGGATCATTACTGTAGTTTCTATCTTGTTGCAAGGTAGCACAAGAATGA
TCTCTGAACTACGATACTATTCAAAAATGTCTTGTGATGCCGGAAAAATGAAT

HrTast2c: Determined nucleotide sequence of RT-PCR product (200 nt)

GGCTCTTGGATTCATCATCGTTCTTATAGCTGGCATTCTTGGTGTAATCATAACTCTTGTACAAGGCAGCGCTAAAAA
ACCTATAGCAGAACAAAGAATGTATGCAAGAATGTCATGTGATGATGAAACGCCAAATTCATTGGAAGTCAATGAATG
CCAAAAGGCAACTTGCATGTTTGACGTCACTACCTGTGCACACG

HrTast2c: Predicted nucleotide sequence from the gene model (Identical sequence between the predicted sequence and determined sequence)

GGCTCTTGGATTCATCATCGTTCTTATAGCTGGCATTCTTGGTGTAATCATAACTCTTGTACAAGGCAGCGCTAAAAA
ACCTATAGCAGAACAAAGAATGTATGCAAGAATGTCATGTGATGATGAAACGCCAAATTCATTGGAAGTCAATGAATG
CCAAAAGGCAACTTGCATGTTTGACGTCACTACCTGTGCACACG

HrTast3c: Determined nucleotide sequence of RT-PCR product (334 nt)

AAGAACCCAAAGCACGTGAGCGCGAAAGCCGGGGATCGCTCTTCAGCTCGAGATAAGAAGAAGCCCGCACAAAACTCA
TCCAACAAAGTTTTGCTCAGCATTGTAGTTTGCGTTGTTCTTGCTGCGGCAATCGGAGCTTTGATGTTTGCTTTATTT
GGAATGTCAACATCGGATTGTCAAAAGTATCTGAAATCTCAAGCTAAAGGCGGCAAAAATAAAGATATTGCGTTAAGT
CCTATTGCGATCTCAGAATGTTTAGAAACCATGTGTACATTTGACGTTCTTCAATGCAACGCCCCATGCCCTTTCGGC
TTTGAAACTTCCCAAGAAAGCG

HrTast3c: Predicted nucleotide sequence from the gene model (Identical sequence between the predicted sequence and determined sequence)

AAGAACCCAAAGCACGTGAGCGCGAAAGCCGGGGATCGCTCTTCAGCTCGAGATAAGAAGAAGCCCGCACAAAACTCA
TCCAACAAAGTTTTGCTCAGCATTGTAGTTTGCGTTGTTCTTGCTGCGGCAATCGGAGCTTTGATGTTTGCTTTATTT
GGAATGTCAACATCGGATTGTCAAAAGTATCTGAAATCTCAAGCTAAAGGCGGCAAAAATAAAGATATTGCGTTAAGT
CCTATTGCGATCTCAGAATGTTTAGAAACCATGTGTACATTTGACGTTCTTCAATGCAACGCCCCATGCCCTTTCGGC
TTTGAAACTTCCCAAGAAAGCG

Figure S2. Determined nucleotide sequences (upper) of the RT–PCR products of the *HrTast1, HrTast2b, HrTast2c* and *HrTast3c* genes and the predicted nucleotide sequence (lower) from the respective gene model.
The yellow and green highlighting indicate the forward and reverse primers, respectively. The purple highlighting indicates the predicted sequence amplified by RT–PCR. The red highlighting indicates the determined nucleotide sequence. The gray highlighted sequence was not detected in the determined sequence, suggesting the presence of an intron region.

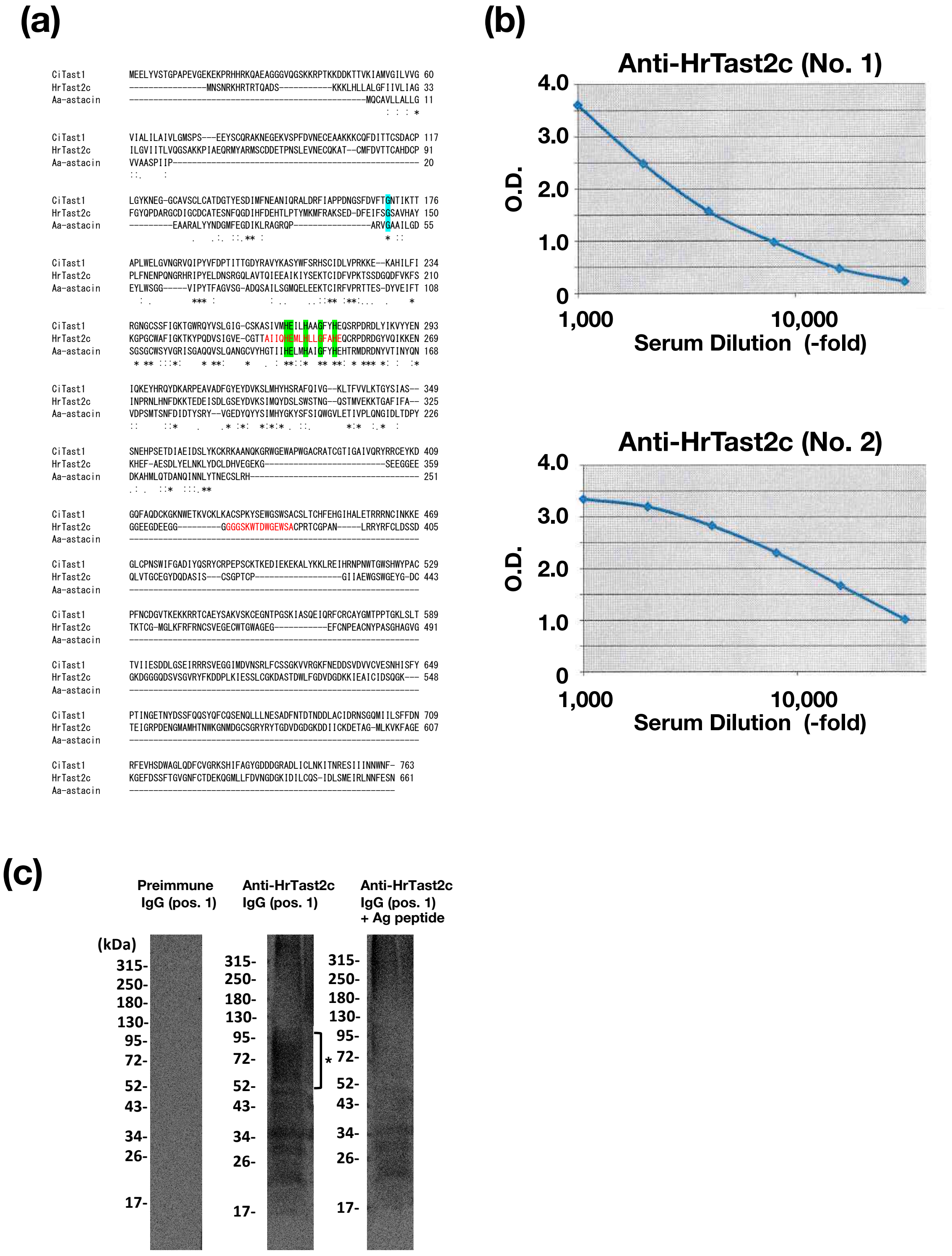

Figure S3. Preparation and Western blotting of HrTast2c peptide antibodies

(a) The amino acid sequence of HrTast2c was aligned with the sequences of *C. intestinalis* CiTast1 and *Astacus astacus* (crayfish) Aa-astacin. The amino acid residues indicated in red correspond to the antigenic peptides (position 1: antigenic peptide region No. 1 (238–254) and position 2 (antigenic peptide region No. 2 (371–384)). The residues highlighted in green are the Zn-binding consensus sites of the metalloprotease. (b) Titers of the antibodies were examined via ELISA. (c) Western blotting of the sperm extract using anti-HrTast2c (position 2) in the presence or absence of the antigenic peptide. The asterisk indicates the specific band of HrTast2c. No appreciable band was observed with anti-HrTast2c IgG (position 1).

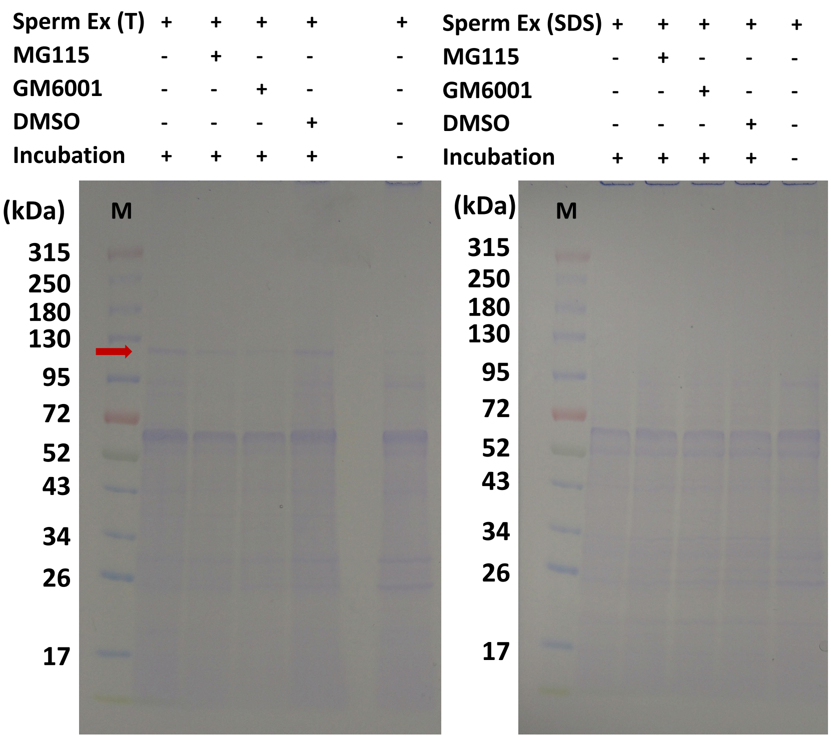

Figure S4. SDS–PAGE of the VC incubated with sperm extract.
A portion (0.2 g) of frozen sperm was thawed, suspended in 1.2 mL of 1% Triton X-100 (or 1% SDS) in 50 mM Tris/HCl (pH of 8.0) and homogenized, followed by centrifugation at 9,000xg for 20 min. The supernatant (sperm extract) was mixed with the isolated VC in the presence or absence of 100 μM GM6001 or MG115, followed by incubation overnight at 13 °C, representing the ideal temperature for *H. roretzi* fertilization. After centrifugation, the precipitated VC was washed repeatedly, subjected to SDS–PAGE under reducing conditions and stained with Coomassie Brilliant Blue R-250. A 120-kDa band of the VC (red arrow) was newly detected after treatment with the sperm extract with 1% Triton X-100 (left panel) but not with 1% SDS (right panel). The appearance of this band was slightly inhibited by GM6001 or MG115 (a proteasome inhibitor). This band was cut from the gel and subjected to LC–MS analysis (Table S1 and Figure S5).

```
1      MRVAALSVCF LLLHAVTARR LHRIRERNSM PVPEDNCEIV QPFDQDYVYT YSYEALTEHE
61     DVSNVLKKAL KISCEVTITV PRRCSGKLRL NDCAIAEAVV SARDEVTYRK LPESEAFDRE
121    MRINELAFHF NGGEITKIFV DKAERTNVTN LKRGILTSLQ VYNPRRPKEF EVGDVYEIPQ
181    VNVYGKGIVE QRILSKEAGF PSSVEIIRDS ARLRLPWERK QWNFIRMRRN DADPFTMAMR
241    NYLAYESENI WSRAKQTCEC SLFRGRQVKN CKCMEYHPME MAHPLLSGLM KGEAVATTIR
300    QSIRVTNTRK MTKKDRSLWD VSRFDEDNIM FEEEWEETNK IYSEGFNAEE TLCYIITEPV
361    NNRFPALVTK LTRWLTTAGQ EDIETAYNRC YGKLSGENKE LARISWVEML NTCINNEWCQ
421    VPYCDSENVT TCENALDSFI RSTTSKEEGV KSAIYRALEA INDPAVTIVE ATLRLCREEA
481    ETTPECVRTL GAVVKNYYAS CEDCERKKPV ENAKKFLMKK LKNDCTASED DEVVSAVIDC
541    IRGLGEKGVF AAEIMLSCGT NPGARNEVAV RALLAYRELT GEVLEKQAET KILATFKNVK
601    NSFEIRLTCF HLLMSKNIEM PVIAEILRPV FEGPEQSALK AHIVEDLRTQ LKLRGTEYCS
661    LHQAVADRVL ECIIKNKWDI GVAYAKLDAK AIERKSEITR EIFIPYLNAR LNCTVKTSLV
721    YTKENAILPV IAKIAVIANI EGKDVTLVEA ALDIVGLKPF LDRLFGENTF YSEFLMKLMN
781    TPMSEINSFT RENSALRLLG EEGRYNYLIN LFRHQTPLYI KLLGYELNLQ DLMNFESIRR
841    MTLANIWKIV DTLEGGFEHE AFGAVRPLMI QHAVPTCLGA PIVSSIDISY VFANKMNLKL
901    NIRKLLFVPN EEAVFDVTPS IYGTAITTRT ILPQFCKSCG TEMEVTVGHN SAIQCNMHTE
961    PSGIRMVIEP KPGKSEILYI KVKRSLIGET GHEPKATTCH QIRWENLSSF QYCYQPVSKS
1021   GLAYELFVEN EEHTEYNVAI SMEGIAQRKA YTKELNINCT VRGRATSEME QAIFSTKFTY
1081   IPAENEVRCS IISDVLKWKV AANYMIYRME GNARGCFVSL NATGLNDLQM MYKNKFMILP
1141   AKENDYIIEG ASEWVGSGLN GGYYMSGKCV EGTEGGNVTR TTDLRVYDKK EPRVSMIVSS
1201   LIVKKIAQTA QIMPGYESRL LVSGAWIPIY KKIEMMMVTM DDARRFEKVM AIRGYFAAGE
1261   TMGPHFYMHR VTGTWKAEGS EEYAASMYAT SNFFHIENSI AYKADGIVTY SKCSFPEEEI
1321   VCGKDMAVSP CVSPCTCEAR TGGSCRCESK CNNGAYVEME WNTVADEKNN RNYSCKIGYN
1381   LWNPELREYS YKKAIASYVN YRECKYCHSF EVTHPKIEIR SITRRSPLHE EKWMKVHYSL
1441   LSYITLYPEV TYFIINHIKN EYFQVGFKVN PINTKTEVTG YKCEGAFFDN LGECNYNCEN
1501   KYSRCKARCE FNLPSIDTSM NTTALFSSSS RPISLDFGIN MVYATLHYDL DGSFTLNERD
1561   RMDCTLKAKY EVYPGEYYEA TAVTRMMNYG SKYNGEGSYT TTRVGAEESQ TVKYSLLSNM
1621   EDSLRYFVVK EICPESETEE PCMSFSINRD GKWLKYDRKL EDNLHDIMTV DSNSEGYKLE
1681   VKMAKEIGMK LKSFRVDLGL ENRYGLNIES EFCNISCWYN AEQGLIAKSN VNTNLINYTM
1741   DATVTSAAVK YNILYNTVRN RGMRMESTMD MKFAIFNAEN PLEIDYRVGI KPGSFGCNLN
1801   MPDLRCMLDI SVEDRLPVER GLKYVLEARV RPQKMIIVNL TLPLGEDSVE ITDTIVGYES
1861   SVDNRLASAY VENINLKWRH ACRAIETTVF TGKCKINSEK ILVNIADGQR SICVADIDFG
1921   YKDPNKHVEL LLNTTMGKLD ILFNVKKEAK NWMMNSKCEI SMVDYVTERM TPVFKIIAEQ
1981   MLAETQPSAN LPDYIFSIMI AHDMHMLTIP KNINVTSRSV AQGARSIGET IAVVDGKIVT
2041   HATGNLIGCH ARFEAFAGYS FCMPLHTDVA MVMEIPLFNS YDHVTCIKTG DSWKFDWISN
2101   ATFPGYALDM DDHFIITSEG DIWKFDYSYR DSIRNAPDRG SKMNCKFDLK YGHCLTQSIF
2161   GINVCLPSTI SIENADVTIS GISELLYAYL PMEFIEFKDI RLQNCEMDYA CSIRGDVVDM
2221   TLKSILRGPS LASPVDVSMK AAFAPNNFEC ATTVMEYDSK FSFGWNPETW GMKITKTMDS
2281   AWYNSNLEIT SPAFMEFSML AFALKTKGAA HDIIPIDINV HYGKANGIWI AKQFCRIASW
2341   EVELNKEAEA YYCKLGCKEY SLLNQTVYMT TTARGRATEC ALAWKSDYVV CKTKFAVEGW
2401   MLEGRKLAVH FPKQECGLEA EYVLNGNSRS CNARIYSREH AEVRCETTEM TAERSFGMMK
2461   CNKQAETGMV NIAIVPVKMS DFPFLRKDSV YDKLKFVTRS ASYVYKTISY DEEALAKDCQ
2521   ETFNCTDQEA CKAMSRSLKT IVSACWKELN RIAGAKIYYL TKPELFAYTI DKWVPGSLNI
2581   PKLSSMMSLF TASERGEITI AHWSFEPYVE AHVGVTMDKT NAFVLHVKAS SEKYSWMDGE
2641   IHLSVMEMEN HTAVNFTHTV YEAMSTIIFD VTESSFILRE VDVSLRGKGV TMKTSGLMQD
2701   GRQTITMTLW EGKTVEYTCL LKTVPTNWKG SEMRCFEGTS EKEVGSIKLG KMSKITEFNS
2761   IDAVLERLFN IENENTRIHI RSTLLNLVKL ATDSHTIEPV NRVSDIAWRS VQYASAWSIH
2821   EAAKMISTTS EMYQIISKFT RNLYNLEPNI GETYERIVEY VGEINLYPEI EKIVINMLTP
2881   LMEFLDRILE GSVSMKTNVE GEELIVEMAL ISARYPFLNC NQVIRLRQHG WNNLTAFAVH
2941   KTRFVRMTAN VGLEKYAFRI IDVMFGLPEV GSNFLQIRFV PDGLGEGIYG VEMVITDSEQ
3001   DQYFCKGGKI SAASPVLHFH CFKFEGTEIP ANPPASSLIA YWDVAIEDVP SIYGSVTGLV
3061   RCVTRLMRVL NACYEDPSTI NELLLKTYHV HDEKIRARLS KTITESCRKW WNEVITSTEG
3121   VLNPEVNKYI KKEGLSVTTV TENLKSYSYD SLYNSLIAEV REPRGAVINI ADLSMIGDIA
3181   SVLFSYSAPI VHYGTGIILM TEVSSSFNAR RLPARSDRAL MLVTRSERLP ILNMEMEVKV
3241   IPDGMINASI SNSLMYSYVL CDIEGALIMP SRAMIGLHHM TESTVEIVFW PEDGTRNPPI
3301   ISCIFGVENR HCCDARFESI VMSTVKCFPY EHGRRGAEVV AKCEISPVEI GTSGAMIVYE
3361   IATGKVDQNR LVDRVLEETC NIKSPEIRKN VITTCERALT TIADYCRDEL INIAKFSNDI
3421   MNATYSHLLE GLVNMFDWSS IVRRAAEGIN VNYLYDLVKD CMDAVVELCS QYSGGVYDVL
3481   EYLYLAGRSL NEICFNFMTY MNESYSTYSL YEWMQLMVAN TATARNCNFG VISTTEYEQA
3541   VTFNNNFYNL PKRVRECDYL LAGDIATNTF SVVLSKNNII INLPGKSLVL DPEMQMYEMR
3601   GSRGEKYEIT LPHFDGHVRC IRTAEHLTCQ TPHVKVIAQA RNGRRFMYSI HVAETVCNKA
3661   RGLMGIGGDT SIVPKWMMMN GKVASTYKEF INSYSTNKAC LIETIPVIPA IEPTPMCKTF
3721   FNSKELNKGV PALHAVLLEA CESVATNTEE ACIYANMYVQ ESLNSGYRAE INPECAVCKN
3781   KQMRAEIERV DVTLMIALSG NTHATRLEEV VTAVRNMANT ERKHYNIRYI TFGGRGEMHH
3841   PYFSVPSGRL VIPAEQPWTL PRFEFNGMKP TIPMIKHCIE FAYRNSKHAV EGVANMFLFV
3901   MPEEMEIDEL IKLPCVENLE KDLAMSYVYI PNACETKGAH ARSPYGVRKL VTKEEICART
3961   PEALATDIAN PYYGRSTTCT CTMEGPFPGM YSDNCKIVA
```

Figure S5. Vitellogenin fragments were detected in the 120-kDa band via SDS–PAGE.
A 120 -kDa band in the SDS–PAGE gel was removed from the gel and subjected to reduced carboxymethylation, trypsin/Lys-C digestion, and LC–MS analysis. The green highlighted sequences were identified as *H. roretzi* vitellogenin fragments (S367.g04226). Sixty-two peptides were identified, which covered 22% of the total sequence of vitellogenin.

Table S1. LC–MS analysis of the 120-kDa protein, which newly appeared after incubation with the isolated VC and sperm extracts *

Checked	Accession	Coverage [%]	# Peptides	# PSMs	# Unique Peptides	# Aas	MW [kDa]	calc. pI	Score Sequest HT: Sequest HT	# Peptides (by Search Engine): Sequest HT	# Protein Groups	Annotation
TRUE	Harore.CG.MTP2014.S563.g04764.01.p	62	57	1058	57	1050	119.2	7.72	3151.29	57	1	2-Oxoglutarate dehydrogenase-like
TRUE	Harore.CG.MTP2014.S367.g04226.01.p	22	62	498	62	3999	453.5	6.24	1503.98	62	1	Vitellinogen, open beta-sheet, Lipid transporter
TRUE	Harore.CG.MTP2014.S135.g10885.02.p	49	37	283	37	1029	114.8	6.8	899.97	37	1	
TRUE	Harore.CG.MTP2014.S647.g10783.01.p	36	29	131	29	1096	119.1	5.8	457.04	29	1	Ankyrin repeat-containing domain superfamily, Ser/Thr-protein phosphatase-6
TRUE	Harore.CG.MTP2014.S2.g03075.01.p	39	31	119	31	1150	129.3	6.37	437.44	31	1	Creatine kinase M-type
TRUE	Harore.CG.MTP2014.S386.g03899.01.p	44	16	95	2	510	57.3	5.06	314.24	16	1	
TRUE	Harore.CG.MTP2014.S336.g01836.01.p	48	16	92	1	467	52.4	4.91	303.59	16	1	
TRUE	Harore.CG.MTP2014.S31.g07310.04.p	36	28	93	0	888	103.5	6.07	299.34	28	1	
TRUE	Harore.CG.MTP2014.S34.g13706.01.p	36	26	97	26	977	111	8.03	288.61	26	1	
TRUE	Harore.CG.MTP2014.S2.g00517.01.p	47	15	81	2	477	52.9	5.11	264.97	15	1	
TRUE	Harore.CG.MTP2014.S35.g03177.01.p	41	13	78	2	446	50.1	4.82	245.01	13	1	
TRUE	Harore.CG.MTP2014.S35.g10317.01.p	47	14	73	3	448	50.3	4.79	241.56	14	1	
TRUE	Harore.CG.MTP2014.S31.g15882.01.p	41	29	81	29	914	105.1	5.31	240.91	29	1	
TRUE	Harore.CG.MTP2014.S184.g10971.01.p	42	13	73	5	453	50.3	5.07	236.65	13	1	
TRUE	Harore.CG.MTP2014.S1.g00695.01.p	16	30	70	30	2619	291	5.08	217.94	30	1	Otoancorin, proteinaceous extracellular matrix
TRUE	Harore.CG.MTP2014.S60.g15555.01.p	30	19	63	19	968	110.4	5.55	214.94	19	1	
TRUE	Harore.CG.MTP2014.S35.g05771.01.p	40	12	63	1	448	50.2	4.79	211.34	12	1	
TRUE	Harore.CG.MTP2014.S31.g07310.01.p	41	19	60	1	596	70	6.28	199.16	19	1	
TRUE	Harore.CG.MTP2014.S13.g15972.01.p	21	20	54	20	1421	161.5	5.82	195.8	20	1	Clathrin heavy chain, N-terminal
TRUE	Harore.CG.MTP2014.S42.g14417.02.p	41	12	60	1	451	50.1	5.06	194.97	12	1	
TRUE	Harore.CG.MTP2014.S422.g05364.01.p	40	12	60	4	400	44.4	5.3	186.04	12	1	
TRUE	Harore.CG.MTP2014.S31.g07310.02.p	42	19	58	1	586	68.9	5.96	184.03	19	1	
TRUE	Harore.CG.MTP2014.S18.g10655.02.p	49	16	49	16	518	55.7	5.68	181.73	16	1	
TRUE	Harore.CG.MTP2014.S250.g09381.01.p	39	11	60	4	408	45.6	6.2	180.35	11	1	
TRUE	Harore.CG.MTP2014.S41.g05014.01.p	24	16	51	16	846	94.2	7.43	179.18	16	1	
TRUE	Harore.CG.MTP2014.S70.g04342.01.p	20	19	49	19	1265	148.2	5.25	167.9	19	1	Myosin head, motor domain
TRUE	Harore.CG.MTP2014.S669.g14984.01.p	31	8	42	8	442	48.3	5.67	153.96	8	1	
TRUE	Harore.CG.MTP2014.S31.g10104.02.p	24	10	45	10	467	51.5	9.28	141.57	10	1	
TRUE	Harore.CG.MTP2014.S41.g10119.01.p	18	11	39	11	1118	126.1	8.59	138.94	11	1	Haem peroxidase domain superfamily, animal type
TRUE	Harore.CG.MTP2014.S746.g04281.01.p	26	12	41	12	650	71.7	5.05	138.04	12	1	
TRUE	Harore.CG.MTP2014.S149.g09374.01.p	18	12	37	12	935	103.3	5.22	131.48	12	1	
TRUE	Harore.CG.MTP2014.S429.g10994.01.p	29	8	34	8	489	54	8.57	127.39	8	1	
TRUE	Harore.CG.MTP2014.S31.g07310.05.p	35	11	38	1	320	36.5	6.96	126.84	11	1	
TRUE	Harore.CG.MTP2014.S528.g00963.01.p	27	14	37	10	657	71.7	5.34	124.9	14	1	
TRUE	Harore.CG.MTP2014.S4.g08850.01.p	27	8	42	1	378	42.2	5.25	123.58	8	1	

TRUE	Harore.CG.MTP2014.S71.g01621.02.p	16	7	39	2	547	59.6	8.28	121.18	7	1	
TRUE	Harore.CG.MTP2014.S237.g03443.01.p	16	15	38	15	1503	169.7	6.43	118.27	15	1	Glycoside hydrolase superfamily
TRUE	Harore.CG.MTP2014.S49.g15467.01.p	17	7	46	1	453	50.6	4.83	118.24	7	1	
TRUE	Harore.CG.MTP2014.S71.g01621.01.p	26	7	35	2	367	39.8	7.59	114.43	7	1	
TRUE	Harore.CG.MTP2014.S156.g08353.02.p	20	14	38	14	996	113.9	5.57	105.48	14	1	
TRUE	Harore.CG.MTP2014.S764.g05199.01.p	12	10	29	10	1192	130.9	5.82	101.2	10	1	P-type ATPase, cytoplasmic domain N
TRUE	Harore.CG.MTP2014.S111.g12265.01.p	25	13	33	13	873	99	5.92	100.4	13	1	
TRUE	Harore.CG.MTP2014.S44.g11791.02.p	7	15	30	15	2668	293.1	7.02	98.18	15	1	HEAT type 2, Stalled ribosome sensor GCN1
TRUE	Harore.CG.MTP2014.S289.g03473.01.p	22	10	33	10	578	63.3	8.72	92.16	10	1	
TRUE	Harore.CG.MTP2014.S321.g06252.02.p	13	10	22	10	1278	146.3	6.74	88.42	10	1	Regulation of nuclear pre-mRNA domain-containing protein 2
TRUE	Harore.CG.MTP2014.S78.g07592.01.p	3	3	28	3	1173	127.9	7.17	86.72	3	1	EGF-like domain, Zona pellucida domain (UNKOWN)
TRUE	Harore.CG.MTP2014.S385.g09733.01.p	5	15	28	15	4558	520.4	6.37	86.54	15	1	Dynein heavy chain domain, Dynein axonemal heavy chain 8
TRUE	Harore.CG.MTP2014.S142.g09627.01.p	11	9	23	9	1262	143.7	7.39	81.53	9	1	Met/Val/Ile-tRNA synthetase, anticodon-binding, Valine--tRNA ligase
TRUE	Harore.CG.MTP2014.S3.g08671.02.p	20	13	27	13	978	110	4.98	81.41	13	1	
TRUE	Harore.CG.MTP2014.S216.g07471.01.p	6	10	21	10	2877	314.4	4.92	77.55	10	1	HrTTSP-1 (Sushi/SCR/CCP domain)
TRUE	Harore.CG.MTP2014.S98.g02137.01.p	9	13	23	13	2326	258.8	6.87	75.02	13	1	Asp/Orn carbamoyltransferase, Multifunctional protein CAD
TRUE	Harore.CG.MTP2014.S124.g10802.01.p	18	11	21	11	1026	115	6.25	73.72	11	1	
TRUE	Harore.CG.MTP2014.S10.g05616.02.p	22	8	26	8	558	61.2	7.88	71.55	8	1	
TRUE	Harore.CG.MTP2014.S614.g07251.02.p	25	9	22	9	479	52.3	8.07	69.83	9	1	
TRUE	Harore.CG.MTP2014.S430.g11250.01.p	5	14	22	14	4786	549.4	6.89	69.42	14	1	Dynein heavy chain, Cytoplasmic dynein 1 heavy chain 1
TRUE	Harore.CG.MTP2014.S113.g02269.02.p	16	10	21	10	895	102.8	4.74	67.69	10	1	
TRUE	Harore.CG.MTP2014.S591.g10684.01.p	21	8	20	8	570	63.3	5.33	67.48	8	1	
TRUE	Harore.CG.MTP2014.S151.g00081.02.p	28	7	18	7	377	41.2	7.96	66.16	7	1	
TRUE	Harore.CG.MTP2014.S458.g12264.01.p	16	5	18	5	519	58.1	7.36	65.74	5	1	
TRUE	Harore.CG.MTP2014.S142.g16064.01.p	17	9	18	9	858	96.7	6.62	62.98	9	1	
TRUE	Harore.CG.MTP2014.S105.g01421.02.p	12	7	21	7	880	97.4	5.05	62.13	7	1	
TRUE	Harore.CG.MTP2014.S110.g15936.02.p	15	7	24	7	765	85	5.52	59.82	7	1	
TRUE	Harore.CG.MTP2014.S354.g07111.01.p	3	9	18	9	4314	495.6	6.18	59.67	9	1	P-loop containing nucleoside triphosphate hydrolase, Dynein heavy chain,
TRUE	Harore.CG.MTP2014.S131.g04501.01.p	7	11	20	11	2506	283.2	7.8	57.22	11	1	Metalloendopeptidase of the mitochondrial matrix , Peptidase M16, C-terminal
TRUE	Harore.CG.MTP2014.S36.g13871.01.p	13	8	20	8	758	87.5	5.02	57.07	8	1	
TRUE	Harore.CG.MTP2014.S298.g08896.01.p	8	8	16	8	1969	220.3	5.81	55.56	8	1	P-loop containing nucleoside triphosphate hydrolase, ABC transporter-like
TRUE	Harore.CG.MTP2014.S261.g08345.01.p	12	6	14	6	696	77	5.9	54.99	6	1	
TRUE	Harore.CG.MTP2014.S12.g12746.01.p	10	7	19	7	902	99	7.08	53.98	7	1	
TRUE	Harore.CG.MTP2014.S44.g01906.01.p	6	5	16	5	1346	150.4	6.96	53.91	5	1	Splicing factor 3B subunit 1
TRUE	Harore.CG.MTP2014.S340.g05164.01.p	13	5	14	5	653	73.2	8.46	52.36	5	1	

TRUE	Harore.CG.MTP2014.S95.g12701.01.p	7	5	14	5	1230	135.4	5.88	50.51	5	1	C-type lectin-like/link domain superfamily, Zona pellucida domain
TRUE	Harore.CG.MTP2014.S429.g10468.02.p	16	6	16	6	453	50.2	7.85	48.42	6	1	
TRUE	Harore.CG.MTP2014.S472.g12698.01.p	11	3	14	3	553	60.8	6.86	47.09	3	1	
TRUE	Harore.CG.MTP2014.S27.g15762.01.p	11	5	12	5	722	81.1	4.91	46.36	5	1	
TRUE	Harore.CG.MTP2014.S1.g03782.02.p	11	5	12	5	778	84.5	6.27	45.86	5	1	
TRUE	Harore.CG.MTP2014.S79.g05222.01.p	18	6	16	6	366	43.2	5.4	44.15	6	1	
TRUE	Harore.CG.MTP2014.S109.g10097.01.p	16	5	11	5	377	42.4	8.16	44.03	5	1	
TRUE	Harore.CG.MTP2014.S147.g10281.01.p	19	8	14	8	734	80.3	6.43	43.2	8	1	
TRUE	Harore.CG.MTP2014.S221.g10564.01.p	17	5	12	5	470	51.8	8.41	41.66	5	1	
TRUE	Harore.CG.MTP2014.S708.g09756.01.p	18	7	13	7	634	68.7	7.83	41.3	7	1	
TRUE	Harore.CG.MTP2014.S140.g03403.01.p	10	7	13	7	1050	115.8	5.99	40.86	7	1	
TRUE	Harore.CG.MTP2014.S1580.g12691.01.p	12	6	11	3	626	68.9	5.27	38.64	6	1	
TRUE	Harore.CG.MTP2014.S4.g10388.01.p	10	7	10	7	1264	135.6	6.24	37.19	7	1	13 EGF-like, conserved site, Zona pellucida domain
TRUE	Harore.CG.MTP2014.S1108.g06775.01.p	10	5	12	4	681	75.1	6.07	36.31	5	1	
TRUE	Harore.CG.MTP2014.S30.g12434.01.p	13	5	11	5	539	59.1	6.02	36.2	5	1	
TRUE	Harore.CG.MTP2014.S107.g13256.01.p	9	4	11	0	636	70.1	5.53	35.89	4	1	
TRUE	Harore.CG.MTP2014.S67.g03499.01.p	13	4	11	4	545	59.2	6.64	35.79	4	1	
TRUE	Harore.CG.MTP2014.S13.g00843.01.p	16	5	11	5	539	58.7	6.76	35.74	5	1	
TRUE	Harore.CG.MTP2014.S41.g15878.01.p	14	4	10	4	536	57.9	7.85	35.06	4	1	
TRUE	Harore.CG.MTP2014.S561.g05685.01.p	22	3	20	3	176	20.1	9.67	34.73	3	1	
TRUE	Harore.CG.MTP2014.S23.g05715.01.p	13	6	10	6	712	79.3	8.25	34.65	6	1	
TRUE	Harore.CG.MTP2014.S60.g02316.01.p	8	5	9	5	1066	119.7	6.4	34.44	5	1	Ubiquitin/SUMO-activating enzyme E1
TRUE	Harore.CG.MTP2014.S316.g06125.01.p	7	2	9	2	315	35.8	6.34	33.76	2	1	
TRUE	Harore.CG.MTP2014.S267.g12320.01.p	22	3	10	3	234	26.9	8.72	33.1	3	1	
TRUE	Harore.CG.MTP2014.S40.g01801.01.p	18	6	11	6	465	51.8	8.47	32.79	6	1	
TRUE	Harore.CG.MTP2014.S67.g02593.01.p	6	4	11	4	937	105.9	8.6	32.76	4	1	
TRUE	Harore.CG.MTP2014.S113.g07932.02.p	14	4	9	4	383	43.1	4.64	32.54	4	1	
TRUE	Harore.CG.MTP2014.S99.g00520.01.p	8	6	9	6	1101	125.8	6.67	32.51	6	1	Importin-beta, N-terminal domain, Armadillo-like helical, Exportin-7
TRUE	Harore.CG.MTP2014.S6.g03382.01.p	14	6	10	6	738	81.2	9.22	31.75	6	1	
TRUE	Harore.CG.MTP2014.S15.g01108.01.p	6	5	10	5	1249	137.7	6.28	31.64	5	1	Aldolase-type TIM barrel, Carbamoyl-phosphate synthetase large subunit-like, Pyruvate carboxylase
TRUE	Harore.CG.MTP2014.S196.g04933.01.p	1	3	9	3	4602	524.9	5.58	31.38	3	1	Dynein axonemal heavy chain, ATPase, dynein-related, AAA domain, LDL receptor class A repeat
TRUE	Harore.CG.MTP2014.S790.g08811.02.p	10	6	9	6	936	106.5	7.01	30.15	6	1	
TRUE	Harore.CG.MTP2014.S75.g01289.01.p	2	7	16	7	4899	553.1	5.8	29.91	7	1	von Willebrand factor, type D domain, Vitellinogen, open beta-sheet
TRUE	Harore.CG.MTP2014.S148.g02958.01.p	19	6	8	6	604	67.2	5.3	28.83	6	1	
TRUE	Harore.CG.MTP2014.S445.g03801.01.p	6	5	9	5	1185	133	6.62	28.71	5	1	

TRUE	Harore.CG.MTP2014.S1.g13663.01.p	3	5	9	5	2144	244.1	5.99	28.16	5	1	AAA+ ATPase domain, Helicase superfamily
TRUE	Harore.CG.MTP2014.S181.g12829.01.p	7	1	17	1	349	40.3	9.55	28.08	1	1	
TRUE	Harore.CG.MTP2014.S28.g00713.01.p	7	5	9	5	1098	120.8	7.42	27.74	5	1	Citrate synthase-like, small alpha subdomain, ATP-citrate synthase
TRUE	Harore.CG.MTP2014.S6.g04858.01.p	9	6	8	6	918	103.9	5.1	27.52	6	1	
TRUE	Harore.CG.MTP2014.S354.g09366.03.p	12	4	7	4	566	62.1	8.32	26.99	4	1	
TRUE	Harore.CG.MTP2014.S331.g12301.01.p	23	3	9	3	220	23.5	5.24	26.62	3	1	
TRUE	Harore.CG.MTP2014.S147.g08883.01.p	8	5	8	5	926	103.6	5.55	26.48	5	1	
TRUE	Harore.CG.MTP2014.S134.g12112.01.p	5	5	7	5	1706	189.9	6.43	26.24	5	1	von Willebrand factor, type A, Inter-alpha-trypsin inhibitor heavy chain H4
TRUE	Harore.CG.MTP2014.S353.g10047.01.p	11	4	7	4	552	59.1	8.05	26.13	4	1	
TRUE	Harore.CG.MTP2014.S308.g08259.01.p	10	3	7	3	526	58.7	5.35	25.64	3	1	
TRUE	Harore.CG.MTP2014.S212.g13755.01.p	10	4	8	4	689	77.7	5.88	25.63	4	1	
TRUE	Harore.CG.MTP2014.S355.g07067.01.p	14	3	8	3	207	23.4	7.11	25.41	3	1	
TRUE	Harore.CG.MTP2014.S627.g10954.01.p	18	5	6	5	770	84.3	8.18	25.4	5	1	
TRUE	Harore.CG.MTP2014.S194.g10300.02.p	8	4	7	4	666	73.1	6.93	25.39	4	1	
TRUE	Harore.CG.MTP2014.S52.g00264.02.p	6	4	10	4	977	109.5	6.23	25.26	4	1	
TRUE	Harore.CG.MTP2014.S14.g03118.02.p	9	3	6	3	631	67.9	6.83	25.18	3	1	
TRUE	Harore.CG.MTP2014.S16.g03171.01.p	16	5	6	5	456	50.2	6.02	24.64	5	1	
TRUE	Harore.CG.MTP2014.S27.g01868.01.p	5	5	8	5	1550	175.3	6.01	24.59	5	1	Nucleotide-diphospho-sugar transferases
TRUE	Harore.CG.MTP2014.S69.g04620.01.p	4	6	7	6	2344	274	8.9	24.55	6	1	
TRUE	Harore.CG.MTP2014.S157.g00824.02.p	16	4	6	4	442	49.8	7.06	24.43	4	1	
TRUE	Harore.CG.MTP2014.S307.g06187.01.p	2	4	7	4	2612	298.1	6.35	24.29	4	1	Centrosomin, N-terminal motif 1
TRUE	Harore.CG.MTP2014.S472.g10985.01.p	14	4	9	4	432	48.2	7.15	24.2	4	1	
TRUE	Harore.CG.MTP2014.S145.g05258.01.p	14	4	7	4	386	43.2	8.16	24.07	4	1	
TRUE	Harore.CG.MTP2014.S332.g13530.02.p	11	4	6	4	553	61.6	6.76	22.47	4	1	
TRUE	Harore.CG.MTP2014.S4.g06929.01.p	7	4	7	4	812	91.2	4.97	22.17	4	1	
TRUE	Harore.CG.MTP2014.S13.g05237.01.p	14	2	5	2	267	30.4	4.97	22.03	2	1	
TRUE	Harore.CG.MTP2014.S486.g04514.01.p	12	4	6	4	535	58.2	6.16	21.22	4	1	
TRUE	Harore.CG.MTP2014.S6.g03931.01.p	15	4	7	4	475	51.2	9.11	20.97	4	1	
TRUE	Harore.CG.MTP2014.S494.g05051.01.p	11	5	6	5	962	109	6.84	20.87	5	1	
TRUE	Harore.CG.MTP2014.S186.g06032.01.p	24	3	6	3	160	18.2	9.91	20.67	3	1	
TRUE	Harore.CG.MTP2014.S123.g15970.01.p	11	4	7	4	541	59.4	6.74	20.29	4	1	
TRUE	Harore.CG.MTP2014.S645.g07698.01.p	5	4	6	4	1139	125.6	4.44	20.18	4	1	SIT4 phosphatase-associated protein family
TRUE	Harore.CG.MTP2014.S383.g06004.01.p	11	3	6	3	444	49.9	8.18	19.91	3	1	
TRUE	Harore.CG.MTP2014.S153.g11623.01.p	7	3	4	3	469	53.4	8.22	19.45	3	1	
TRUE	Harore.CG.MTP2014.S330.g00890.01.p	7	3	6	2	585	66	7.05	19.38	3	1	
TRUE	Harore.CG.MTP2014.S136.g10424.01.p	3	3	5	3	1763	201.2	6.1	18.73	3	1	CH-like domain in sperm protein, Sperm flagellar protein 2
TRUE	Harore.CG.MTP2014.S371.g01082.01.p	12	3	5	3	400	43.6	6.7	17.83	3	1	

TRUE	Harore.CG.MTP2014.S75.g08394.01.p	8	5	5	5	1029	111.6	7.12	17.18	5	1	
TRUE	Harore.CG.MTP2014.S80.g01333.01.p	5	4	7	4	1272	143.3	7.4	17.01	4	1	WD40 repeat, Coatomer subunit alpha
TRUE	Harore.CG.MTP2014.S157.g02180.01.p	8	4	7	4	811	90.2	8.47	16.86	4	1	
TRUE	Harore.CG.MTP2014.S450.g08886.01.p	14	3	5	3	265	30.3	4.88	16.79	3	1	
TRUE	Harore.CG.MTP2014.S16.g10824.01.p	4	5	5	5	1686	190	6.04	16.73	5	1	
TRUE	Harore.CG.MTP2014.S79.g09933.01.p	13	3	6	3	336	35.6	9.06	16.54	3	1	
TRUE	Harore.CG.MTP2014.S77.g11516.01.p	16	4	6	4	506	54.8	7.36	16.42	4	1	
TRUE	Harore.CG.MTP2014.S280.g07136.01.p	11	3	5	3	538	60.5	6.4	16.29	3	1	
TRUE	Harore.CG.MTP2014.S43.g08812.01.p	1	1	6	1	1780	201.5	5.39	16.2	1	1	IQ motif, EF-hand binding site, Leu-rich repeat domain superfamily
TRUE	Harore.CG.MTP2014.S214.g10227.01.p	6	2	5	2	539	59.3	7.84	16.09	2	1	
TRUE	Harore.CG.MTP2014.S465.g09135.01.p	2	4	6	4	2610	290.7	6.38	16.07	4	1	Ankyrin repeat-containing domain superfamily
TRUE	Harore.CG.MTP2014.S6.g10660.01.p	17	3	6	3	307	34.1	8.63	15.95	3	1	
TRUE	Harore.CG.MTP2014.S19.g14776.01.p	14	3	4	3	344	35.8	7.96	15.8	3	1	
TRUE	Harore.CG.MTP2014.S186.g08439.01.p	4	3	5	3	763	86	6.07	15.57	3	1	
TRUE	Harore.CG.MTP2014.S28.g12475.01.p	2	2	5	2	1178	130.9	5.33	15.46	2	1	WD40/YVTN repeat-like-containing domain superfamily
TRUE	Harore.CG.MTP2014.S147.g10997.01.p	3	1	6	1	517	54.7	7.88	15.23	1	1	
TRUE	Harore.CG.MTP2014.S583.g05089.01.p	7	4	4	4	875	98.6	5.45	15.14	4	1	
TRUE	Harore.CG.MTP2014.S46.g04753.02.p	6	2	4	2	544	59.6	6.67	15.12	2	1	
TRUE	Harore.CG.MTP2014.S571.g10074.01.p	5	3	4	3	916	102.6	6.74	14.8	3	1	
TRUE	Harore.CG.MTP2014.S391.g06117.01.p	9	3	5	3	558	60	6.89	14.77	3	1	
TRUE	Harore.CG.MTP2014.S67.g10005.01.p	3	3	5	3	1206	136.6	9.04	14.74	3	1	Myosin head, motor domain
TRUE	Harore.CG.MTP2014.S42.g05517.01.p	6	3	4	3	863	96	6.33	14.68	3	1	
TRUE	Harore.CG.MTP2014.S333.g10767.03.p	10	3	5	3	434	48.5	6.04	14.3	3	1	
TRUE	Harore.CG.MTP2014.S423.g09913.01.p	7	3	5	3	625	68.3	6.7	14.07	3	1	
TRUE	Harore.CG.MTP2014.S18.g02500.01.p	5	3	4	3	1056	121.1	4.88	14.03	3	1	Armadillo-like helical, Importin-beta, N-terminal domain
TRUE	Harore.CG.MTP2014.S261.g06577.01.p	4	2	7	2	813	91.1	6.28	13.82	2	1	HrTast3c
TRUE	Harore.CG.MTP2014.S1111.g02890.01.p	4	3	4	3	1037	110.9	4.48	13.69	3	1	EGF-like, conserved site, Zona pellucida domain
TRUE	Harore.CG.MTP2014.S195.g02243.01.p	8	3	4	3	587	67.3	6.68	13.51	3	1	
TRUE	Harore.CG.MTP2014.S847.g02600.01.p	10	2	4	2	274	30.2	9.64	13.47	2	1	
TRUE	Harore.CG.MTP2014.S103.g09461.02.p	4	1	3	1	508	56.1	5.48	13.24	1	1	
TRUE	Harore.CG.MTP2014.S156.g05923.01.p	5	2	4	2	533	59.1	8.16	13.13	2	1	
TRUE	Harore.CG.MTP2014.S30.g10399.01.p	6	2	4	2	552	59.1	7.39	13.12	2	1	
TRUE	Harore.CG.MTP2014.S224.g01430.01.p	7	3	4	3	612	69.6	6.21	12.63	3	1	
TRUE	Harore.CG.MTP2014.S67.g07046.01.p	7	3	4	3	508	55.3	6.92	12.51	3	1	
TRUE	Harore.CG.MTP2014.S831.g04134.01.p	3	2	4	2	826	94.7	6.2	12.49	2	1	
TRUE	Harore.CG.MTP2014.S767.g02331.01.p	6	1	4	1	225	25.4	10.67	12.24	1	1	
TRUE	Harore.CG.MTP2014.S525.g03800.01.p	2	3	3	3	2942	325.1	5.59	12.16	3	1	P-loop containing nucleoside triphosphate hydrolase, Immunoglobulin-like fold

TRUE	Harore.CG.MTP2014.S4.g01451.01.p	2	1	4	1	827	92.9	5.19	12.13	1	1	
TRUE	Harore.CG.MTP2014.S23.g12563.01.p	5	2	5	2	540	60.1	7.69	12.1	2	1	
TRUE	Harore.CG.MTP2014.S110.g10196.01.p	6	1	3	1	265	29.6	5.45	11.96	1	1	
TRUE	Harore.CG.MTP2014.S746.g13749.01.p	5	3	3	3	892	102.1	6.65	11.9	3	1	
TRUE	Harore.CG.MTP2014.S108.g02133.01.p	9	3	3	3	531	58.9	7.05	11.84	3	1	
TRUE	Harore.CG.MTP2014.S162.g10534.01.p	7	3	5	3	550	59.7	6.06	11.69	3	1	
TRUE	Harore.CG.MTP2014.S64.g10372.01.p	19	2	4	2	171	19.8	10.58	11.47	2	1	
TRUE	Harore.CG.MTP2014.S47.g10422.01.p	7	2	3	2	471	51.3	7.87	11.44	2	1	
TRUE	Harore.CG.MTP2014.S35.g01689.01.p	5	2	3	2	708	80.1	7.17	11.44	2	1	
TRUE	Harore.CG.MTP2014.S30.g03976.01.p	9	2	7	2	268	30.4	9.77	11.43	2	1	
TRUE	Harore.CG.MTP2014.S103.g12155.01.p	9	2	3	2	302	34.1	6.89	11.42	2	1	
TRUE	Harore.CG.MTP2014.S41.g06549.01.p	4	2	6	2	661	72.8	4.87	11.38	2	1	HrTast2c
TRUE	Harore.CG.MTP2014.S50.g04071.01.p	11	3	3	3	421	48.6	6.99	11.17	3	1	
TRUE	Harore.CG.MTP2014.S236.g11437.03.p	3	2	3	2	1310	146.2	5.86	11.13	2	1	Armadillo-like helical, TATA-binding protein interacting (TIP20)
TRUE	Harore.CG.MTP2014.S46.g14756.02.p	4	2	4	2	575	62.8	6.9	11.1	2	1	
TRUE	Harore.CG.MTP2014.S523.g09804.01.p	2	2	3	2	1466	167.6	8.09	11.09	2	1	Sushi/SCR/CCP superfamily, Zona pellucida domain
TRUE	Harore.CG.MTP2014.S5.g14725.01.p	12	2	3	2	244	28.7	10.35	10.87	2	1	
TRUE	Harore.CG.MTP2014.S205.g08933.01.p	7	2	4	2	345	38.3	7.8	10.72	2	1	
TRUE	Harore.CG.MTP2014.S100.g05627.01.p	6	2	3	2	556	62.8	6.01	10.64	2	1	
TRUE	Harore.CG.MTP2014.S23.g02817.02.p	5	1	2	1	348	40.5	5.05	10.19	1	1	
TRUE	Harore.CG.MTP2014.S6.g12905.01.p	4	2	3	2	695	76.1	6.05	10.13	2	1	
TRUE	Harore.CG.MTP2014.S7.g05985.02.p	7	2	3	2	449	47.9	8.15	10.01	2	1	
TRUE	Harore.CG.MTP2014.S92.g13373.01.p	7	3	6	3	742	82.4	7.24	10	3	1	
TRUE	Harore.CG.MTP2014.S621.g13981.01.p	10	2	3	2	327	35.8	8.02	9.97	2	1	
TRUE	Harore.CG.MTP2014.S36.g05846.01.p	7	2	5	2	388	42.8	8.62	9.9	2	1	
TRUE	Harore.CG.MTP2014.S298.g05232.01.p	4	2	3	2	1131	124.3	7.02	9.84	2	1	Zinc finger, Sec23/Sec24-type, von Willebrand factor A-like domain superfamily
TRUE	Harore.CG.MTP2014.S85.g11614.01.p	5	1	2	1	408	44.6	5.26	9.77	1	1	
TRUE	Harore.CG.MTP2014.S22.g06725.01.p	6	3	3	3	736	85.2	7.53	9.63	3	1	
TRUE	Harore.CG.MTP2014.S15.g13541.01.p	7	2	3	2	412	45.4	7.17	9.57	2	1	
TRUE	Harore.CG.MTP2014.S90.g01676.01.p	21	2	4	2	103	11.3	11.36	9.55	2	1	
TRUE	Harore.CG.MTP2014.S150.g09769.01.p	4	2	3	2	603	66.4	7.01	9.4	2	1	
TRUE	Harore.CG.MTP2014.S43.g09856.01.p	6	2	4	2	708	79.6	6.79	9.32	2	1	
TRUE	Harore.CG.MTP2014.S413.g15056.01.p	15	2	3	2	155	18.1	10.76	9.28	2	1	
TRUE	Harore.CG.MTP2014.S223.g11286.01.p	12	2	3	2	221	24.6	7.3	9.06	2	1	
TRUE	Harore.CG.MTP2014.S82.g02718.01.p	7	1	3	1	248	28.3	10.43	9.04	1	1	
TRUE	Harore.CG.MTP2014.S205.g08426.01.p	11	1	2	1	137	15.6	11.15	8.9	1	1	
TRUE	Harore.CG.MTP2014.S110.g06301.01.p	4	2	3	2	756	85.5	6.84	8.83	2	1	

TRUE	Harore.CG.MTP2014.S502.g15320.01.p	6	2	3	2	482	54	8.53	8.69	2	1	
TRUE	Harore.CG.MTP2014.S30.g01378.01.p	1	2	2	2	2792	320.8	5.24	8.65	2	1	CH domain superfamily, Calponin homology domain
TRUE	Harore.CG.MTP2014.S106.g01066.01.p	9	1	3	1	136	16	10.92	8.58	1	1	
TRUE	Harore.CG.MTP2014.S84.g13805.01.p	7	2	2	2	512	58.5	5.4	8.53	2	1	
TRUE	Harore.CG.MTP2014.S149.g13454.01.p	1	2	2	2	2701	300.8	7.72	8.42	2	1	CCR4-Not complex component, Not1, C-terminal, MIF4G-like domain superfamily
TRUE	Harore.CG.MTP2014.S24.g01199.01.p	1	2	3	2	3607	408.3	5.38	8.32	2	1	NACHT nucleoside triphosphatase, von Willebrand factor A-like domain superfamily
TRUE	Harore.CG.MTP2014.S383.g09914.01.p	26	2	2	2	116	13	9.07	7.97	2	1	
TRUE	Harore.CG.MTP2014.S445.g09290.01.p	3	1	2	1	467	53.2	5.68	7.93	1	1	
TRUE	Harore.CG.MTP2014.S95.g08963.01.p	6	2	2	2	522	59.5	8.24	7.92	2	1	
TRUE	Harore.CG.MTP2014.S46.g10726.01.p	20	2	2	2	140	15.7	7.4	7.89	2	1	
TRUE	Harore.CG.MTP2014.S1007.g12818.01.p	2	1	3	1	779	86.4	5.92	7.87	1	1	
TRUE	Harore.CG.MTP2014.S141.g08538.01.p	3	2	3	2	727	81	8.46	7.79	2	1	
TRUE	Harore.CG.MTP2014.S305.g10083.01.p	3	2	2	2	1147	128.1	8.19	7.77	2	1	Zona pellucida domain
TRUE	Harore.CG.MTP2014.S737.g07016.01.p	3	1	3	1	415	46.3	10.02	7.74	1	1	
TRUE	Harore.CG.MTP2014.S172.g06608.02.p	5	2	2	2	619	68.8	5.91	7.71	2	1	
TRUE	Harore.CG.MTP2014.S68.g01484.01.p	8	2	3	2	596	68.2	6.74	7.4	2	1	
TRUE	Harore.CG.MTP2014.S34.g15643.01.p	13	1	2	1	115	13.3	11.03	7.37	1	1	
TRUE	Harore.CG.MTP2014.S140.g11429.01.p	4	1	2	1	364	40.8	5.21	7.33	1	1	
TRUE	Harore.CG.MTP2014.S140.g01661.01.p	3	2	2	2	1193	130.5	6.87	7.31	2	1	WD40 repeat
TRUE	Harore.CG.MTP2014.S631.g12579.01.p	3	2	2	2	1154	129.3	6.79	7.02	2	1	von Willebrand factor A-like domain superfamily
TRUE	Harore.CG.MTP2014.S30.g01044.02.p	2	1	2	1	725	82.4	8.25	6.97	1	1	
TRUE	Harore.CG.MTP2014.S450.g01203.01.p	6	1	2	1	261	30.3	4.75	6.94	1	1	
TRUE	Harore.CG.MTP2014.S146.g14145.01.p	6	2	2	2	403	45.9	6.6	6.91	2	1	
TRUE	Harore.CG.MTP2014.S56.g01213.01.p	3	2	3	2	946	105.6	6.11	6.89	2	1	
TRUE	Harore.CG.MTP2014.S575.g02915.01.p	14	2	2	2	187	22	5.07	6.85	2	1	
TRUE	Harore.CG.MTP2014.S71.g06759.02.p	2	1	2	1	935	100.9	7.77	6.83	1	1	
TRUE	Harore.CG.MTP2014.S157.g15980.01.p	1	1	2	1	1289	148.3	5.1	6.71	1	1	EF-hand domain
TRUE	Harore.CG.MTP2014.S372.g10597.01.p	5	1	3	1	369	42.2	5.64	6.71	1	1	
TRUE	Harore.CG.MTP2014.S3.g11362.01.p	6	1	2	1	420	47.7	7.05	6.62	1	1	
TRUE	Harore.CG.MTP2014.S101.g04847.01.p	5	1	2	1	476	52.9	6.96	6.59	1	1	
TRUE	Harore.CG.MTP2014.S15.g10121.01.p	3	2	2	2	948	105.2	7.2	6.54	2	1	
TRUE	Harore.CG.MTP2014.S161.g12646.01.p	2	2	2	2	1192	134.4	6.06	6.53	2	1	Six-hairpin glycosidase-like superfamily
TRUE	Harore.CG.MTP2014.S14.g01603.01.p	2	1	2	1	777	89	4.97	6.52	1	1	
TRUE	Harore.CG.MTP2014.S326.g00898.01.p	1	1	2	1	1253	138.8	6.24	6.4	1	1	P-type ATPase, A domain superfamily, Cation-transporting P-type ATPase, N-terminal
TRUE	Harore.CG.MTP2014.S127.g14166.01.p	11	1	2	1	125	13.8	8.57	6.37	1	1	
TRUE	Harore.CG.MTP2014.S168.g02673.01.p	2	1	3	1	482	53.3	5.01	6.36	1	1	

TRUE	Harore.CG.MTP2014.S8.g14446.01.p	10	2	2	2	326	36.7	9.74	6.35	2	1	
TRUE	Harore.CG.MTP2014.S419.g01234.01.p	1	2	2	2	2323	261.9	6.38	6.23	2	1	WD40-repeat-containing domain superfamily, RZZ complex, subunit KNTC1/ROD, C-terminal
TRUE	Harore.CG.MTP2014.S48.g14248.01.p	2	1	2	1	679	75.5	4.65	6.19	1	1	
TRUE	Harore.CG.MTP2014.S304.g11637.01.p	11	1	2	1	193	22.3	10.02	6.17	1	1	
TRUE	Harore.CG.MTP2014.S3.g13829.01.p	1	1	2	1	1184	135.1	5.72	6.16	1	1	Cysteine-rich Golgi apparatus protein 1 repeat, eukaryote
TRUE	Harore.CG.MTP2014.S267.g11175.01.p	7	1	2	1	200	22.8	6.21	6.16	1	1	
TRUE	Harore.CG.MTP2014.S107.g06022.01.p	1	1	2	1	1105	123.8	6.54	6.07	1	1	Armadillo-like helical, MMS19, C-terminal
TRUE	Harore.CG.MTP2014.S832.g14609.01.p	9	2	2	2	328	37.8	6.62	6.03	2	1	
TRUE	Harore.CG.MTP2014.S2.g09887.01.p	8	1	2	1	161	17.7	6.62	5.93	1	1	
TRUE	Harore.CG.MTP2014.S16.g09648.03.p	4	1	2	1	492	55.5	6.48	5.9	1	1	
TRUE	Harore.CG.MTP2014.S891.g06367.01.p	3	1	2	1	627	71.6	5.17	5.87	1	1	
TRUE	Harore.CG.MTP2014.S374.g05972.02.p	5	2	2	2	608	68.6	7.15	5.83	2	1	
TRUE	Harore.CG.MTP2014.S208.g09216.01.p	6	1	2	1	177	20.7	11.08	5.82	1	1	
TRUE	Harore.CG.MTP2014.S119.g00635.01.p	1	1	2	1	3362	373.1	9.48	5.68	1	1	BAT2, N-terminal, Protein PRRC2
TRUE	Harore.CG.MTP2014.S33.g11458.01.p	5	1	1	1	368	43.1	9.25	5.64	1	1	
TRUE	Harore.CG.MTP2014.S90.g05849.01.p	8	1	2	1	139	15.9	10.23	5.63	1	1	
TRUE	Harore.CG.MTP2014.S87.g03995.01.p	3	1	2	1	440	48.6	7.81	5.43	1	1	
TRUE	Harore.CG.MTP2014.S835.g15832.01.p	4	1	2	1	274	29.9	10.29	5.42	1	1	
TRUE	Harore.CG.MTP2014.S449.g08835.02.p	2	1	2	1	646	70.4	6.62	5.4	1	1	
TRUE	Harore.CG.MTP2014.S413.g09530.02.p	1	1	2	1	904	103.3	9.77	5.32	1	1	
TRUE	Harore.CG.MTP2014.S63.g10260.01.p	3	1	3	1	451	51.2	7.78	5.32	1	1	
TRUE	Harore.CG.MTP2014.S311.g02959.01.p	3	1	2	1	390	42.3	5.85	5.3	1	1	
TRUE	Harore.CG.MTP2014.S1.g13128.01.p	11	1	2	1	168	19.4	10.04	5.23	1	1	
TRUE	Harore.CG.MTP2014.S214.g09308.01.p	2	1	2	1	722	79	4.87	5.2	1	1	
TRUE	Harore.CG.MTP2014.S429.g09491.02.p	2	1	2	1	663	76.2	6.33	5.07	1	1	
TRUE	Harore.CG.MTP2014.S623.g08848.01.p	6	1	2	1	308	33.6	7.56	4.98	1	1	
TRUE	Harore.CG.MTP2014.S4.g00773.01.p	4	1	5	1	290	33.4	10.86	4.94	1	1	
TRUE	Harore.CG.MTP2014.S26.g12147.01.p	0	1	2	1	5894	682	8.78	4.84	1	1	
TRUE	Harore.CG.MTP2014.S650.g08219.01.p	3	1	2	1	478	55.6	6.77	4.72	1	1	
TRUE	Harore.CG.MTP2014.S125.g04129.01.p	2	1	3	1	892	102.1	8.47	4.69	1	1	
TRUE	Harore.CG.MTP2014.S31.g02867.01.p	5	1	1	1	348	39.7	10.04	4.56	1	1	
TRUE	Harore.CG.MTP2014.S340.g06256.01.p	18	1	1	1	120	13.6	5.29	4.52	1	1	
TRUE	Harore.CG.MTP2014.S74.g15677.01.p	2	1	2	1	388	43.8	4.87	4.51	1	1	
TRUE	Harore.CG.MTP2014.S372.g15251.01.p	2	1	2	1	818	92.1	8.12	4.41	1	1	
TRUE	Harore.CG.MTP2014.S13.g10960.01.p	5	1	1	1	362	41.2	6.84	4.33	1	1	
TRUE	Harore.CG.MTP2014.S130.g12323.01.p	6	1	1	1	713	77.6	7.56	4.31	1	1	
TRUE	Harore.CG.MTP2014.S414.g10728.01.p	8	1	1	1	274	31.5	7.46	4.3	1	1	

TRUE	Harore.CG.MTP2014.S385.g07417.01.p	5	1	2	1	746	82.4	8.12	4.23	1	1	
TRUE	Harore.CG.MTP2014.S7.g02985.01.p	3	1	1	1	503	55.8	8.81	4.23	1	1	
TRUE	Harore.CG.MTP2014.S50.g03819.02.p	4	1	1	1	547	62.1	8.18	4.2	1	1	
TRUE	Harore.CG.MTP2014.S60.g16068.01.p	3	1	1	1	679	76.3	6.77	4.19	1	1	
TRUE	Harore.CG.MTP2014.S118.g15652.01.p	2	1	1	1	850	93.3	7.8	4.17	1	1	
TRUE	Harore.CG.MTP2014.S123.g02789.02.p	4	1	1	1	442	49.6	5.14	4.13	1	1	
TRUE	Harore.CG.MTP2014.S149.g16046.01.p	4	1	1	1	515	57.8	9.5	4.09	1	1	
TRUE	Harore.CG.MTP2014.S31.g14580.01.p	3	1	3	1	535	60.3	8.18	4.08	1	1	
TRUE	Harore.CG.MTP2014.S117.g03302.01.p	1	1	1	1	1465	169.1	6.25	4.01	1	1	
TRUE	Harore.CG.MTP2014.S126.g01852.01.p	0	1	1	1	21269	2354.9	5.82	4	1	1	Not accesible
TRUE	Harore.CG.MTP2014.S1776.g14135.01.p	5	1	1	1	256	28	5.3	3.97	1	1	
TRUE	Harore.CG.MTP2014.S15.g02198.01.p	1	1	1	1	2707	302.3	8.57	3.94	1	1	Zinc finger, FYVE/PHD-type、Rab-binding domain
TRUE	Harore.CG.MTP2014.S4.g00229.01.p	3	1	1	1	522	57.6	5.72	3.93	1	1	
TRUE	Harore.CG.MTP2014.S64.g12694.01.p	8	1	3	1	547	62.3	8.47	3.91	1	1	
TRUE	Harore.CG.MTP2014.S299.g05035.01.p	2	1	1	1	888	97.3	7.02	3.89	1	1	
TRUE	Harore.CG.MTP2014.S89.g00434.01.p	3	1	1	1	695	79.6	5.8	3.87	1	1	
TRUE	Harore.CG.MTP2014.S288.g14328.01.p	3	1	1	1	577	68.8	7.18	3.82	1	1	
TRUE	Harore.CG.MTP2014.S56.g05509.01.p	5	1	1	1	300	33.5	8.81	3.81	1	1	
TRUE	Harore.CG.MTP2014.S247.g14709.01.p	2	1	2	1	870	94.2	8.76	3.81	1	1	
TRUE	Harore.CG.MTP2014.S110.g15936.04.p	12	1	1	1	182	20.9	8.88	3.79	1	1	
TRUE	Harore.CG.MTP2014.S243.g08637.01.p	3	1	1	1	1161	129.8	8.32	3.79	1	1	Tyr-protein kinase, active site、Ser-thr/tyr-protein kinase, ATP binding site
TRUE	Harore.CG.MTP2014.S60.g06435.01.p	1	1	1	1	1691	187.1	5.02	3.77	1	1	Laminin EGF domain
TRUE	Harore.CG.MTP2014.S226.g09901.01.p	4	1	1	1	622	69.7	4.92	3.76	1	1	
TRUE	Harore.CG.MTP2014.S21.g13223.01.p	2	1	2	1	1327	147.5	6.96	3.76	1	1	
TRUE	Harore.CG.MTP2014.S168.g12138.01.p	11	1	1	1	140	14.7	10.58	3.73	1	1	
TRUE	Harore.CG.MTP2014.S371.g11506.01.p	1	1	1	1	1559	174	6.65	3.71	1	1	
TRUE	Harore.CG.MTP2014.S509.g02694.01.p	1	1	1	1	1470	164.1	6.24	3.67	1	1	
TRUE	Harore.CG.MTP2014.S168.g09155.02.p	5	1	1	1	313	34.8	6.2	3.66	1	1	
TRUE	Harore.CG.MTP2014.S76.g13401.01.p	3	1	1	1	887	102	9.38	3.66	1	1	
TRUE	Harore.CG.MTP2014.S10.g02106.01.p	4	1	2	1	512	55.4	8.03	3.66	1	1	
TRUE	Harore.CG.MTP2014.S73.g01831.01.p	3	1	1	1	744	84.1	8.13	3.65	1	1	
TRUE	Harore.CG.MTP2014.S47.g15565.01.p	2	1	1	1	700	79.9	4.86	3.64	1	1	
TRUE	Harore.CG.MTP2014.S74.g07562.01.p	3	1	1	1	946	107.8	6.27	3.62	1	1	
TRUE	Harore.CG.MTP2014.S534.g09325.01.p	4	1	2	1	739	82.9	8.75	3.61	1	1	
TRUE	Harore.CG.MTP2014.S93.g14844.01.p	7	1	1	1	452	51.1	6.15	3.6	1	1	
TRUE	Harore.CG.MTP2014.S75.g07902.01.p	4	1	1	1	582	65.4	8.63	3.6	1	1	
TRUE	Harore.CG.MTP2014.S149.g09326.01.p	1	1	1	1	1017	113.7	4.96	3.56	1	1	

TRUE	Harore.CG.MTP2014.S1046.g02016.01.p	2	1	1	1	726	83.4	5.71	3.55	1	1	
TRUE	Harore.CG.MTP2014.S2.g15152.02.p	3	1	1	1	604	68.4	8.44	3.54	1	1	
TRUE	Harore.CG.MTP2014.S244.g04568.01.p	5	1	1	1	338	37.3	6.71	3.5	1	1	
TRUE	Harore.CG.MTP2014.S118.g14167.01.p	4	1	2	1	451	49.6	7.08	3.49	1	1	
TRUE	Harore.CG.MTP2014.S175.g04271.01.p	0	1	1	1	4538	498.1	5.05	3.47	1	1	
TRUE	Harore.CG.MTP2014.S628.g01969.01.p	2	1	1	1	736	81.5	7.15	3.47	1	1	
TRUE	Harore.CG.MTP2014.S28.g10387.01.p	4	1	1	1	422	48.8	7.02	3.47	1	1	
TRUE	Harore.CG.MTP2014.S122.g00894.01.p	5	1	1	1	283	32.7	7.75	3.45	1	1	
TRUE	Harore.CG.MTP2014.S311.g06071.01.p	8	1	2	1	153	17.5	11.08	3.44	1	1	
TRUE	Harore.CG.MTP2014.S461.g12874.01.p	2	1	1	1	690	75	5.44	3.43	1	1	
TRUE	Harore.CG.MTP2014.S679.g05466.01.p	2	1	1	1	660	75	8.13	3.4	1	1	
TRUE	Harore.CG.MTP2014.S183.g02724.02.p	3	1	1	1	490	55.2	7.84	3.36	1	1	
TRUE	Harore.CG.MTP2014.S143.g04060.01.p	1	1	1	1	1148	127.6	5.16	3.35	1	1	
TRUE	Harore.CG.MTP2014.S205.g15409.03.p	5	1	1	1	319	36.7	5.41	3.31	1	1	
TRUE	Harore.CG.MTP2014.S2.g11886.03.p	1	1	1	1	1273	144	7.28	3.3	1	1	
TRUE	Harore.CG.MTP2014.S6.g03763.01.p	1	1	2	1	2617	297.8	6.01	3.3	1	1	
TRUE	Harore.CG.MTP2014.S387.g09369.02.p	2	1	1	1	1006	112	6.2	3.29	1	1	
TRUE	Harore.CG.MTP2014.S306.g02139.01.p	4	1	1	1	319	35.5	4.83	3.29	1	1	
TRUE	Harore.CG.MTP2014.S76.g11452.01.p	2	1	1	1	946	108.3	6.4	3.28	1	1	
TRUE	Harore.CG.MTP2014.S192.g13237.01.p	5	1	1	1	274	30.6	9.28	3.26	1	1	
TRUE	Harore.CG.MTP2014.S669.g10298.01.p	3	1	1	1	389	45.7	5.73	3.24	1	1	
TRUE	Harore.CG.MTP2014.S17.g08304.01.p	5	1	1	1	295	32.4	8.47	3.24	1	1	
TRUE	Harore.CG.MTP2014.S458.g05196.01.p	15	1	1	1	239	27.6	7.61	3.23	1	1	
TRUE	Harore.CG.MTP2014.S7.g12019.01.p	6	1	1	1	287	31.1	5.87	3.21	1	1	
TRUE	Harore.CG.MTP2014.S38.g00174.01.p	2	1	1	1	819	91	5.1	3.19	1	1	
TRUE	Harore.CG.MTP2014.S15.g15019.01.p	6	1	1	1	305	34.8	8.57	3.17	1	1	
TRUE	Harore.CG.MTP2014.S7.g09104.01.p	1	1	1	1	2524	288.9	5.3	3.13	1	1	
TRUE	Harore.CG.MTP2014.S73.g08441.01.p	2	1	3	1	770	85	8.54	3.13	1	1	
TRUE	Harore.CG.MTP2014.S607.g00966.02.p	3	1	1	1	484	56.1	6.49	3.12	1	1	
TRUE	Harore.CG.MTP2014.S368.g05207.01.p	0	1	1	1	3268	364.3	5.94	3.09	1	1	
TRUE	Harore.CG.MTP2014.S48.g11671.01.p	3	1	2	1	514	59.1	5.12	3.09	1	1	
TRUE	Harore.CG.MTP2014.S121.g01710.02.p	3	1	1	1	558	60.4	8.27	3.07	1	1	
TRUE	Harore.CG.MTP2014.S188.g00623.01.p	8	1	1	1	186	21.7	11.75	3.07	1	1	
TRUE	Harore.CG.MTP2014.S390.g07331.01.p	2	1	1	1	969	105.9	6.81	3.06	1	1	
TRUE	Harore.CG.MTP2014.S226.g13679.01.p	1	1	1	1	1357	153.8	6.89	3.05	1	1	
TRUE	Harore.CG.MTP2014.S1.g04124.01.p	3	1	1	1	472	53.2	5.96	3.04	1	1	
TRUE	Harore.CG.MTP2014.S62.g10547.01.p	1	1	1	1	746	84.1	5.71	3.03	1	1	
TRUE	Harore.CG.MTP2014.S1082.g06650.01.p	3	1	1	1	450	50.6	8.43	3.02	1	1	

TRUE	Harore.CG.MTP2014.S91.g12135.01.p	1	1	3	1	721	83.9	5.96	3.01	1	1	
TRUE	Harore.CG.MTP2014.S38.g11303.01.p	3	1	1	1	444	48.1	8.37	3	1	1	
TRUE	Harore.CG.MTP2014.S222.g13084.01.p	8	1	1	1	159	18.4	10.26	2.99	1	1	
TRUE	Harore.CG.MTP2014.S447.g06021.01.p	1	1	1	1	1257	145.8	6.79	2.98	1	1	
TRUE	Harore.CG.MTP2014.S140.g12641.01.p	3	1	1	1	357	40.5	8.34	2.97	1	1	
TRUE	Harore.CG.MTP2014.S19.g03035.01.p	3	1	1	1	415	47.6	6.92	2.97	1	1	
TRUE	Harore.CG.MTP2014.S446.g10162.01.p	4	1	1	1	366	41.6	8.35	2.96	1	1	
TRUE	Harore.CG.MTP2014.S27.g08265.01.p	2	1	1	1	729	82.6	6.24	2.96	1	1	
TRUE	Harore.CG.MTP2014.S28.g12910.01.p	1	1	1	1	1902	217.3	7.21	2.96	1	1	
TRUE	Harore.CG.MTP2014.S226.g05546.01.p	2	1	1	1	819	90.8	5.68	2.94	1	1	
TRUE	Harore.CG.MTP2014.S100.g02713.01.p	2	1	1	1	489	54.4	7.99	2.94	1	1	
TRUE	Harore.CG.MTP2014.S79.g03111.01.p	2	1	1	1	553	60	6.87	2.94	1	1	
TRUE	Harore.CG.MTP2014.S138.g03243.01.p	0	1	1	1	23333	2586.8	6.39	2.94	1	1	
TRUE	Harore.CG.MTP2014.S258.g05422.01.p	8	1	2	1	216	24.9	6.55	2.93	1	1	
TRUE	Harore.CG.MTP2014.S856.g11039.01.p	8	1	1	1	151	16.7	8.78	2.93	1	1	
TRUE	Harore.CG.MTP2014.S140.g12867.01.p	4	1	1	1	388	44	8.32	2.92	1	1	
TRUE	Harore.CG.MTP2014.S216.g11302.01.p	2	1	1	1	734	84.1	7.08	2.92	1	1	
TRUE	Harore.CG.MTP2014.S62.g02057.02.p	2	1	1	1	752	85.1	5.27	2.92	1	1	
TRUE	Harore.CG.MTP2014.S106.g15906.01.p	2	1	1	1	686	74.9	9.01	2.91	1	1	
TRUE	Harore.CG.MTP2014.S74.g13908.01.p	5	1	2	1	255	27.1	4.6	2.9	1	1	
TRUE	Harore.CG.MTP2014.S329.g06332.01.p	1	1	1	1	871	102.3	7.4	2.9	1	1	
TRUE	Harore.CG.MTP2014.S3.g01965.01.p	3	1	1	1	472	50.9	6.79	2.89	1	1	
TRUE	Harore.CG.MTP2014.S326.g07218.01.p	2	1	1	1	546	61.5	4.94	2.89	1	1	
TRUE	Harore.CG.MTP2014.S346.g00379.01.p	1	1	1	1	1663	185.3	6.54	2.88	1	1	
TRUE	Harore.CG.MTP2014.S270.g05292.02.p	1	1	1	1	1009	115.9	6.64	2.88	1	1	
TRUE	Harore.CG.MTP2014.S431.g11829.01.p	2	1	1	1	840	93	8.65	2.86	1	1	
TRUE	Harore.CG.MTP2014.S56.g00817.01.p	2	1	1	1	760	85.4	8.24	2.85	1	1	
TRUE	Harore.CG.MTP2014.S217.g01139.01.p	2	1	1	1	822	93	5.63	2.84	1	1	
TRUE	Harore.CG.MTP2014.S575.g14321.01.p	3	1	1	1	514	56.2	6.67	2.84	1	1	
TRUE	Harore.CG.MTP2014.S108.g14969.01.p	4	1	1	1	376	42.4	7.08	2.83	1	1	
TRUE	Harore.CG.MTP2014.S217.g03510.01.p	2	1	1	1	496	54.5	6.24	2.83	1	1	
TRUE	Harore.CG.MTP2014.S288.g06079.01.p	5	1	1	1	208	23.9	10.36	2.83	1	1	
TRUE	Harore.CG.MTP2014.S134.g09823.01.p	1	1	2	1	1104	122.2	7.36	2.83	1	1	
TRUE	Harore.CG.MTP2014.S917.g14209.01.p	6	1	2	1	483	57	8.68	0	1	1	
TRUE	Harore.CG.MTP2014.S74.g00446.01.p	5	1	3	1	537	59.8	8.41	0	1	1	
TRUE	Harore.CG.MTP2014.S8.g05712.01.p	6	1	1	1	651	73.1	7.06	0	1	1	
TRUE	Harore.CG.MTP2014.S190.g04310.01.p	2	1	3	1	1781	199.7	9.17	0	1	1	
TRUE	Harore.CG.MTP2014.S16.g15062.01.p	7	1	3	1	390	44.2	9.23	0	1	1	

TRUE	Harore.CG.MTP2014 .S18.g11724.01.p	5	1	2	1	642	72.9	5.52	0	1	1	
TRUE	Harore.CG.MTP2014 .S68.g08098.01.p	4	1	6	1	697	77.3	4.68	0	1	1	

* A 120-kDa VC band on SDS–PAGE, which appeared after incubation of the VC with the sperm extract, was cut from the gel and subjected to reduced carboxymethylation, trypsin and Lys–C digestion and LC–MS analysis via an Orbitrap Fusion Lumos Tribrid mass spectrometer (Thermo Fisher Scientific, CA, USA) connected to an UltiMate 3000 RSLCnano System (Thermo Fisher Scientific). The analytical column was a reversed-phase column (PepMap RSLC C18, 0.075 × 150 mm; Thermo Fisher Scientific). The mobile phase was composed of 0.1% formic acid (FA) solution (buffer A) and acetonitrile containing 0.1% FA (buffer B). The flow rate was set to 300 µl/min, and the gradient program was as follows: an isocratic flow at 0% B for 5 min and a linear gradient from 0% B to 40% B for 145 min. Full mass spectra and MS/MS spectra were acquired using an Orbitrap and an ion trap, respectively. The spectral data obtained via MS/MS were used for database search analysis with the Sequest HT algorithm (Proteome Discoverer 2.4 SP1, Thermo Fisher Scientific) via protein data encoded from the *H. roretzi* genome (Harore.MTP2014.protein.fasta, ANISEED database [30]).

Proteins with a molecular mass greater than 110 kDa (red) and with more than a single unique peptide are annotated in the right column, where possible VC proteins and sperm proteins are indicated by purple and red letters, respectively. Notably, two fragments of HrTast2c and HrTast3c were also detected (yellow), suggesting the existence of the proteins in the mixture of the VC and sperm extract. HrTTSP-1 (314.4 kDa) indicated by yellow is a sperm membrane protein, which is capable of binding to HrVC70, a sperm receptor [20, 21].
